# Targeted metabolomic profiling reveals inflammation–associated longitudinal changes in plasma metabolites following on-pump coronary bypass surgery

**DOI:** 10.3389/fmed.2025.1673132

**Published:** 2025-10-30

**Authors:** Frieder Neu, Max Wacker, Sven Schuchardt, Sam Varghese, George Awad, Fakhar H. Waqas, Jens Wippermann, Frank Pessler, Priya Veluswamy

**Affiliations:** ^1^Research Group Biomarkers for Infectious Diseases, TWINCORE Centre for Experimental and Clinical Infection Research, a Joint Venture Between Hannover Medical School and the Helmholtz Centre for Infection Research, Braunschweig, Germany; ^2^Department of Cardiothoracic Surgery, Heart Surgery Research, Otto-von-Guericke University Hospital, Magdeburg, Germany; ^3^Department of Bio and Environmental Analytics, Fraunhofer Institute for Toxicology and Environmental Medicine, Hannover, Germany; ^4^Centre for Individualised Infection Medicine, Hannover, Germany

**Keywords:** bile acids, biomarkers, coronary artery disease, coronary artery bypass graft, free fatty acids, metabolomics, phosphatidylcholine, plasma metabolites

## Abstract

**Aims:**

Cardiac surgery leads to major post-operative changes in metabolism, but their exact nature and the underlying risk factors remains obscure. We aimed to characterize changes in plasma metabolites after coronary artery bypass grafting (CABG) to identify intra- and post-operative risk factors for global and specific alterations in plasma metabolites post-operatively.

**Methods:**

We performed a targeted metabolomic screen on plasma samples from patients undergoing on-pump CABG for coronary artery disease (CAD), collected 1 day before and 1, 3, and 7 days after surgery. We assessed correlations with parameters of intra-operative course (cardiopulmonary bypass time and aortic cross-clamping time), intensive care unit (ICU) care, (length of ICU stay, duration of mechanical ventilation, duration of epinephrine/dobutamine or norepinephrine therapy), and systemic inflammation.

**Results:**

Out of 1,019 detectable analytes, 970 passed the quality screen and were included in the analysis. With respect to d0, the greatest degree of change in metabolite populations occurred by d1, but substantial changes persisted through d7. Metabolites could be classified into those which were predominantly downregulated (e.g., triglycerides, bile acids, cholesterol esters, lysophosphatidylcholines, indoles and derivatives), up- or downregulated (e.g., phosphatidylinositol, phosphatidylethanolamines, phosphatidic acids, ceramides), or upregulated (free fatty acids, monoglycerides). Concentrations of food- and/or microbiota-derived metabolites (indole derivatives, trimethylamine N-oxide, trigonelline) were markedly reduced, particularly on d1 and d3. Changes in metabolite concentrations correlated most strongly with plasma C-reactive protein concentration (*r* = −0.67 to 0.59) and blood leukocyte count (−0.63 to 0.32) and less with intra-operative (−0.62 to 0.50) and ICU care (−0.52 to 0.38) parameters. Of note, neither C-reactive protein (CRP) nor leukocyte count correlated significantly with an intra-operative or ICU parameter.

**Conclusions:**

These results reveal pronounced changes in plasma metabolite populations after CABG, which likely result from the combined effects of surgical and post-operative stress, systemic inflammation, reduced dietary intake, and possibly changes in gut microflora.

## Introduction

Despite advances in interventional therapies and medication, coronary artery bypass grafting (CABG) is still globally considered the gold standard for clinically significant coronary artery disease (CAD) (1). This complex surgical procedure is usually conducted with cardiopulmonary bypass (CPB) using the heart-lung-machine (HLM). The major role of HLM is to support heart and lung function, maintaining blood circulation and oxygenation while heart beat is stopped for the duration of surgery. This is achieved by de-routing the blood through the tubes and membranes of the HLM (2), where intimate contact between blood components (cellular and plasmatic fractions) and the non-endothelial surfaces of CPB and a subsequent post-CPB reperfusion injury could lead to aberrant blood cellular responses, i.e., by generating reactive oxygen and nitrogen species (3). Such responses are associated with increased post-surgical systemic inflammation with a high likelihood of organ dysfunction. Furthermore, traumatic incisions made during the surgical procedure are also considered key factors for acute systemic inflammation among these patients (4). Although both traumatic surgical injury and CPB have been known to induce conventional pathological factors including complements, endotoxin release, cytokines, oxygen-free radicals, and platelet-activating factors, changes in small-molecule metabolite populations are now being increasingly appreciated as manifestations of distinct inflammatory endotypes (5).

Metabolic signatures in CAD provide rapid and systematic identification of small-molecule metabolites and distinct lipid metabolites, which are considered as “fingerprint” of an individual, at the time of sampling. Indeed, altered cellular metabolism due to an underlying atherosclerotic pathological condition in CAD has been clearly evidenced by metabolic profiling in blood-based fluids (6). Plasma lipidomics includes profiling of sterol lipids, glycerolipids, glycerophospholipids, and sphingolipids, which are considered the most abundant lipids (7). Of note, both phosphatidylcholines (PC) and lysophosphatidylcholines (LPC) are important members of glycerophospholipids, where PC are integral molecules for (i) maintaining the structure of cell membrane and (ii) circulating lipoproteins and natural surfactants, and LPC is a hydrolyzed bioactive catabolite of PC. Both molecules play crucial roles in atherosclerosis development and progression (8).

Bile acids (BAs) are emerging as key metabolites in the context of cardiovascular health and disease, where they are originally synthesized in the liver and further metabolized by gut microbiota (9). These BAs play pivotal roles in lipid metabolism, as well as in cardiovascular physiology upon signaling through the bile salt receptor [the farnesoid-X receptor (FXR)] (10). The BA-FXR signaling axis is known for its anti-atherosclerotic properties, by regulating lipid profiles and vascular tension, and thereby showing protective effects during atherogenesis (11, 12). Nevertheless, dysregulated BA levels could also contribute to cardiovascular pathologies (13). This complexity is further underscored by research linking BAs with gut microbiota in the regulation of myocardial function (14). Despite this, the exact mechanisms remain poorly understood, with some studies indicating that altered bile acid profiles, particularly the balance between primary and secondary BAs, may influence cardiac outcomes in heart disease (15). Furthermore, clinical data suggest that serum concentration of BAs is inversely correlated with the presence of CAD and myocardial infarction (16), highlighting the dual role of BAs in both protection and injury of cardiac tissue.

Likewise, tryptophan (Trp) metabolism plays a significant role in cardiovascular physiology, where kynurenine, one of the Trp catabolites which breaks down further into several biologically active metabolites (17), is now considered a biomarker for coronary artery disease (18, 19). Furthermore, Trp is also readily catabolized by intestinal microbiota to produce indole and derivatives thereof, including indoleacetic acid (3-IAA), indolealdehyde (I3A), indolepropionic acid (3-IPA), and indole sulfate (Ind-SO_4_) (20). Of note, indole derivatives are ligands for the aryl hydrocarbon receptor (AhR), and AhR signaling plays important roles in maintaining vascular homeostasis (21). Most of these microbial-generated indole derivatives possess beneficial roles by (i) promoting tolerance; (ii) regulating mucosal integrity and gut permeability; (iii) promoting anticoagulant properties; (iv) ameliorating atherogenesis, showing their cardioprotective effects (22). In contrast, indole sulfate accumulates as uremic toxin and therefore has a detrimental role in patients with vascular disease comorbid with chronic kidney failure (23, 24). Elevated levels of indole sulfate are involved in the pathogenesis of aortic arterial stiffness among CAD patients (25). Though many studies show a causal relationship between blood metabolites and CAD progression, there are still major gaps in knowledge in identifying prominent metabolic signatures during surgery-related acute stress. In particular, there are no longitudinal studies assessing changes in peripheral blood metabolites after on-pump CABG surgery. Metabolites with significantly altered concentrations post-operative could potentially serve to predict postsurgical adverse events and CAD outcome.

We therefore investigated the impact of cardiac surgery and the consequences of using CPB, on plasma metabolite alterations among CAD patients. Here, we analyzed CAD patients before surgery and followed them for three time points post CABG, to observe a possible link between alterations in plasma metabolites and systemic acute inflammation. Furthermore, we focused on associations between metabolic changes and several perioperative parameters including (i) CPB utilized time; (ii) aortic cross clamping (ACC) time; (iii) mechanical ventilation (MV) time; (iv) epinephrine/dobutamine (E/dob) time; and (v) norepinephrine (NE) time. The results suggest that these metabolic indicators could serve as valuable molecular tools to identify disturbances in key metabolic pathways among CAD patients who have undergone CABG.

## Materials and methods

### Study cohort

This prospective study was approved by the Ethics Committee of Otto-von-Guericke-University Hospital (OVGU), Magdeburg, Germany (file no. 183/19), which is in accordance with the ethical principles of the Declaration of Helsinki. After giving informed consent, CAD patients number (*N*) = 24 from the Department of Cardiothoracic Surgery at OVGU were enrolled in 2023, all undergoing elective on-pump CABG. Among them, *n* = 3 (12.5%) had two vessel disease, and *n* = 21 (87.5%) had three vessel disease. The study did not include single vessel CAD patients. Inclusion criteria were (i) adults >18 years of age and (ii) elective on-pump CABG. Exclusion criteria were (i) intake of immunosuppressive medication, (ii) viral hepatitis or HIV infection, and (iii) anemia with hemoglobin < 7 mmol/L. All patients underwent the same standardized protocol for anesthesia (≥8 h no food and ≥2 h no beverage intake before surgery, general anesthesia with propofol, rocuronium and sufentanil), perfusion, and surgery. All patients resumed oral food intake on post-operative day 1 except for two patients who developed a complicated clinical course.

### Study design

Blood samples were collected at the following time points: 1 day before surgery (pre-operative; d0, *n* = 24), 1 day post-surgery (post-operative; d1, *n* = 24), 3 days post-surgery (d3, *n* = 24), and either six (*n* = 10) or 7 days post-surgery (d7; *n* = 10). The 10 d6 CAD patients were sampled on day 6 because they were to be discharged to rehabilitation later that day. A two-group comparison between day 6 and 7 post-operative showed no significant separation in the principal component analysis (PCA) based on metabolic differences, and a differential abundance analysis [fold change (FC) > |1.5|, false discovery rate (FDR) < 0.05] did not reveal any significantly changed metabolites (Supplementary Figure S1). Thus, samples from day 6 and 7 post-operative were collectively analyzed as day 7 (d7, *n* = 20). On day 7, metabolite data were not available for four patients as one patient died on day 6 due to sudden cardiac death after transfer to general ward, and the blood sample for metabolomic studies was not taken from the other three patients due to internal error during sample collection. A PCA based on metabolite differences between d1 and d3 vs. d0 showed that the metabolite patterns of the four patients with missing samples on d7 did not differ from those with a complete set of samples on d7 (Supplementary Figure S2), and the missing data were therefore treated as missing at random and not replaced by imputation. However metabolic data from these 4 patients for day 1 and day 3 were included in the comparisons against day 0. The data were paired, and each post-operative sample (d1, d3, d7) was compared to the pre-operative sample (d0) from the same patient.

### Blood and clinical parameters

Plasma samples were obtained from venous blood and used for metabolite profiling. Blood was centrifuged to sediment cells and debris, and aliquots of supernatants were frozen at −80 °C within 2 h. Additionally, the following laboratory parameters were measured at each time point (d0, d1, d3 and d7) at the central laboratory of OVGU hospital using standard protocols: leukocyte count, C-reactive protein (CRP), alanine aminotransferase (ALAT), creatine kinase (CK), creatinine, prothrombin time, international normalized ratio (INR), and activated partial thrombin time (aPTT). Furthermore, the recorded surgical parameters comprised number of vessel bypasses, cardiopulmonary bypass time (CPB time), and ACC time. The intensive care unit (ICU) parameters that were recorded comprised length of ICU stay, time of total MV, and length of catecholamine therapy, (E/Dob time and NE time).

### Targeted metabolomic profiling

Metabolite concentrations were measured on a SCIEX 5500 QTrapTM mass spectrometer (SCIEX, Darmstadt, Germany) using the MxP Quant 500XL kit (Biocrates, Life Sciences AG, Innsbruck, Austria). The kit combines flow injection analysis tandem mass spectrometry (FIA-MS/MS) for lipids and liquid chromatography tandem mass spectrometry (LC-MS/MS) using Agilent 1290 Infinity II liquid chromatography (Santa Clara, CA, USA) coupled with a tandem mass spectrometer for small molecules. Depending on the sample to be analyzed, this kit allows the quantification of up to 1,019 metabolites, where analyte classes (number of metabolites) are described as follows: alkaloids (1), amine oxides (AO; 1), amino acids (AA; 20), amino acid related molecules (AAR; 30), bile acids (BA; 14), biogenic amines (BioAm; 9), carboxylic acids (CA; 7), cresols (1), fatty acids (FA; 12), hormones and related metabolites (4), indoles and derivatives (ID; 4), nucleobases and related metabolites (2), vitamins and cofactors (1), carbohydrates and related metabolites (1), acylcarnitines (AC; 40), lysophosphatidic acids (LPA; 8), phosphatidic acids (PA; 41), lysophosphatidylcholines (LPC; 12), phosphatidylcholines (PC; 76), lysophosphatidylethanolamines (LPE; 43), phosphatidylethanolamines (PE; 95), lysophophatidylglycerols (LPG; 10), phosphatidylglycerols (PG; 64), lysophosphatidylinositols (LPI; 16), phosphatidylinositols (PI; 53), lysophosphatidylserines (LPS; 12), phosphatidylserines (PS; 18), sphinganines and sphingosines (SPBP; 8), sphinganine and sphingosine phosphates (SPBP; 8), sphingomyelines (SM; 15), ceramides (Cer; 29), dihydroceramides (8), glycosylceramides (34), cholesterol esters (CE; 22), monoglycerides (MG; 12), diglycerides (DG; 44), and triglycerides (TG; 242). Metabolite extraction and all analytical assays were conducted in accordance with the protocols provided by the manufacturer (https://biocrates.com/mxp-quant-500-xl/, accessed on 02 Mai 2024). The WebIDQ™ software tool (Biocrates Life Science AG, Innsbruck, Austria) was used for peak integration and to calculate metabolite concentrations.

### Quality screen

The samples were measured on three 96 well plates. Plate-to-plate variability was addressed by target normalization [median Quality Control level 2]. At least 4 QC2 were distributed and analyzed on each plate. To assess metabolic changes as accurately as possible, all metabolites were included in the analyses except for those with a constant value across all samples in the respective comparison. 51, 49 and 52 metabolites were excluded when comparing d1 vs. d0, d3 vs. d0 and d7 vs. d0, respectively. Supplementary Table S1 shows a complete list of included metabolites in each comparison. Any concentrations that were measured below limit of detection (LOD) were replaced with the pseudovalue LOD/2.

### Statistical analyses

Due to non-normal distribution of the data, nonparametric statistical tests were employed. For PCA, data were log10 transformed to correct for heteroscedasticity and auto-scaled to make all variables equally important (26). Statistical Analysis of Metabolomics Data. University of North Carolina at Charlotte; Department of Bioinformatics & Genomics. Retrieved April 21, 2024, from https://www.uab.edu/proteomics/metabolomics/workshop/2014/statistical%20analysis.pdf. Mean Euclidian distances were calculated for each metabolite class based on the first two principal components of the PCAs in the respective comparison to assess the metabolite classes with the greatest degree of change. Differential abundance analysis was performed using the Wilcoxon rank sum test to assess significance of differences between groups based on paired samples. The Benjamini-Hochberg correction was implemented with a false discovery rate (FDR) of 0.05 to account for multiple testing. For paired analysis, fold change (FC) was determined by calculating the ratio between paired samples, resulting in one FC per pair. The means of these FC values, pair means, was then computed. A FC threshold of ≥|1.5| was set for differential abundance analysis. Spearman's rank correlation coefficient was used to assess correlations. PCA, differential abundance analysis, and correlation testing were performed using the open-source software MetaboAnalyst 5.0 (https://www.metaboanalyst.ca/). Values for standard parameters that were < LOD were replaced by LOD/2: i.e., CRP LOD = 0.6 mg/L, was replaced with 0.3 mg/L.

## Results

### Recruitment and characteristics of CAD patients

Demographic and clinical characteristics of the patient cohort are summarized in Tables 1, 2. There was the expected preponderance of male patients and high proportion of patients with comorbidities which confer an increased cardiac risk (diabetes, overweight, chronic obstructive pulmonary disease, COPD; Table 1). Most patients presented for bypass surgery for three or more coronary vessels, and the recorded values for bypass time, need for vasopressors, and length of ICU stay fell into the expected range. Postoperatively, there was a transient increase in creatine kinase (indicating cardiac ischemia), but renal and liver function parameters remained stable (Table 2). There were no significant sex-specific differences except a higher blood leukocyte count in women on post-operative day 3 [12.1 (10.1–14.8) vs. 9.6 (7.13–13.5), *p* = 0.024]. Median CRP values rose greatly, manifesting the typical peak on day 3 and subsequent decline, but the median value of 59.5 mg/L on d7 indicated ongoing systemic inflammation. In contrast, the marked leukocytosis on d1 essentially normalized by d7. Taken together, these data suggest that the patient cohort has the typical clinical characteristics of individuals presenting for elective coronary bypass graft surgery and manifests the expected postoperative evolution of inflammatory parameters.

**Table 1:** Baseline and intra-operative characteristics.

**Demographic characteristics**	
Gender female, *n* (%)	8 (33.3)
Age at first blood collection, years [median (range))	66 (49–82)
BMI [median (range)]	28.2 (23.1–39.7)
Acute coronary syndrome, *n* (%)	4 (16.7)
LVEF, % [median (range)]	55 (18–65)
**Comorbidities**	
Diabetes mellitus (I or II), *n* (%)	12 (50)
Smoker, *n* (%)	8 (33.3)
COPD, *n* (%)	2 (8.0)
Chronic kidney disease (eGFR < 90 ml/min/1.73 m^2^), *n* (%)	18 (75)
Atrial fibrillation, *n* (%)	4 (16.7)
**Surgical procedure**	
Double bypass, *n* (%)	2 (8.2)
Triple bypass, *n* (%)	11 (45.8)
> triple bypass, *n* (%)	11 (45.8)
**Surgical parameters**	
Cardiopulmonary bypass time, min [median (range)]	83.5 (46–166)
Aortic cross clamping time, min [median (range)]	51 (32–81)
Operating time, min [median (range)]	200 (105–315)

**Table 2:** Hematologic and clinical chemistry parameters.

**Parameter**	**d0**	**d1**	**d3**	**d7**	**Reference values**
**Median (range)**					
Leukocyte count (Gpt/L)	7.7 (5.8–93.8)	13.4 (5.2–17.7)	10.7 (7.1–19)	9 (3.2–20)	3.9–10.4
CRP (mg/L)	1.4 (0.3–67.1)	32.7 (13.8–114.0)	131.3 (58.6–242.5)	59.5 (22.7–169.1)	< 5
ALAT (μmol/s.L)	0.5 (0.3–1.0)	0.4 (0.3–2.5)	0.5 (0.2–1.5)	0.6 (0.0–1.6)	0.17–0.58
Creatin kinase (μmol/L)	2.2 (0.5–10.5)	8.2 (3.4–74.2)	4.8 (0.8–35.4)	2.1 (0.6–18.0)	< 0.40
Creatinine (μmol/L)	80.5 (60.0–232.0)	73.5 (0.4–227.0)	78 (57.0–252.0)	79.5 (59.0–234.0)	45–84
CKD-EPI (ml/min/1.73 m^2^)	1.4 (0.4–9.3)	1.5 (0.4–23.3)	1.4 (0.2–1.8)	1.3 (0.2–1.9)	≥1.5
Quick (%)	102.0 (76.0–120.0)	84.0 (32.3–110.0)	87.0 (73.0–104.0)	83.5 (74.0–96.0)	>70
INR	1.0 (0.4–10.0)	1.1 (1.0–1.4)	1.1 (1.0–1.2)	1.1 (1.0–1.2)	< 1.15
aPTT (s)	28.7 (25.7–34.2)	27.8 (19.4–32.9)	28.8 (24.6–39.7)	29.4 (23.3–58.0)	< 34.4

### Postoperative plasma metabolite profiles show pronounced shifts over time

**Figure 1:**
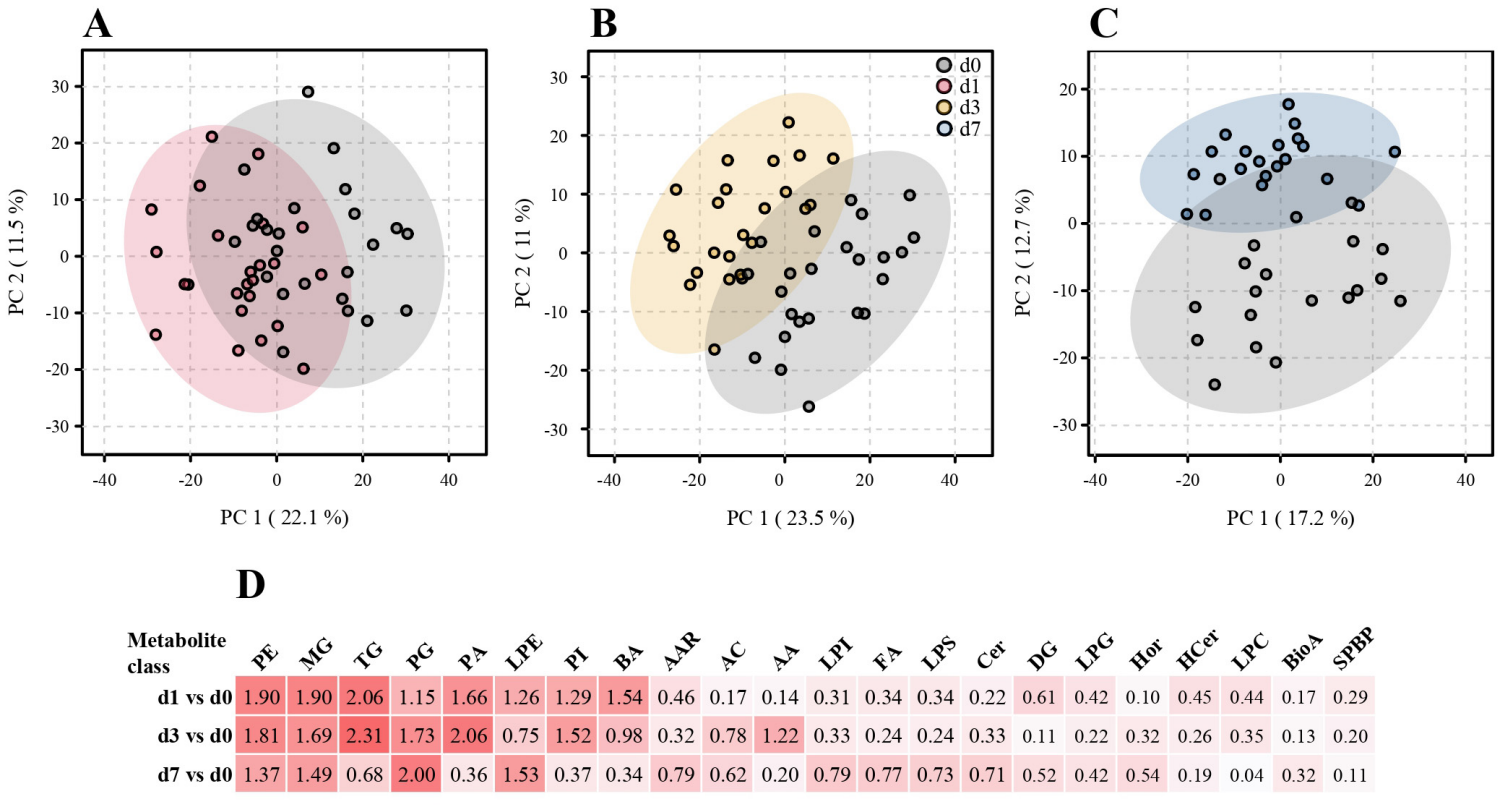
Global and class-specific changes in metabolite populations at the three time points post-op. A principal component analysis (PCA) was performed using 968, 970 and 967 metabolites that passed quality assessment for the comparisons d1 vs. d0, d3 vs. d0 and d7 vs. d0, respectively (Supplementary Table S1). The degree of reprogramming within individual metabolite classes was additionally assessed by Euclidian distance analysis. Total patient numbers (*N*) = 24 with four time points/patient, where *n* = 24 (d0); 24 (d1); 24 (d3); 20 (d7). **(A–C)** PCA; **(D)** Euclidian distance analysis. A complete list of Euclidian distances across all metabolite classes and the abbreviations used is contained in Supplementary Table S3.

A PCA was performed to assess global differences in metabolite populations at each of the three post-operative time points (d1, d3, d7) with respect to the preoperative samples (d0). While substantial separation was already evident on d1, it increased further by d3 and persisted through d7. However, variance shifted from PC1 to PC2 on d7, indicating that it was driven, in part, by different metabolites than at the earlier time points. At all three time points, the greatest degree of variance with respect to d0 was due to alterations in triglyceride (TG) concentrations. For instance, the 20 analytes with the greatest contribution to variance in the first component (PC1, which contributed 17%−23.5% of overall variance) were TGs at all time points, indicating a considerable degree of coregulation within this analyte class (Figures 1A–C, Supplementary Table S2). Regarding PC2, PA and PG contributed most to variance at all time points, peaking on d3 and decreasing on d7. TG impacted on PC2 only on d1. MG and LPEs contributed to variance in PC2 only on d7, largely explaining the separation between d0 and d7 samples along PC2 at this time point.

Euclidian distance analysis supported the above notion that the greatest changes in metabolite populations occurred by d1 and d3, whereby the greatest changes were seen in LPE, PE, TG, MG, PC, PA, LPC, and bile acids (Figure 1D). The latter stood out in that there was a marked increase in variance on d1, which decreased by d3 and essentially normalized by d7. Nonetheless, several metabolite classes showed the largest Euclidian distance on d7. These were characterized by, overall, moderate Euclidian distances on d1 and d3 (e.g., LPI, FA, LPS, Cer), underscoring the differences in metabolite populations on day 7 compared to d1 and d3.

**Figure 2:**
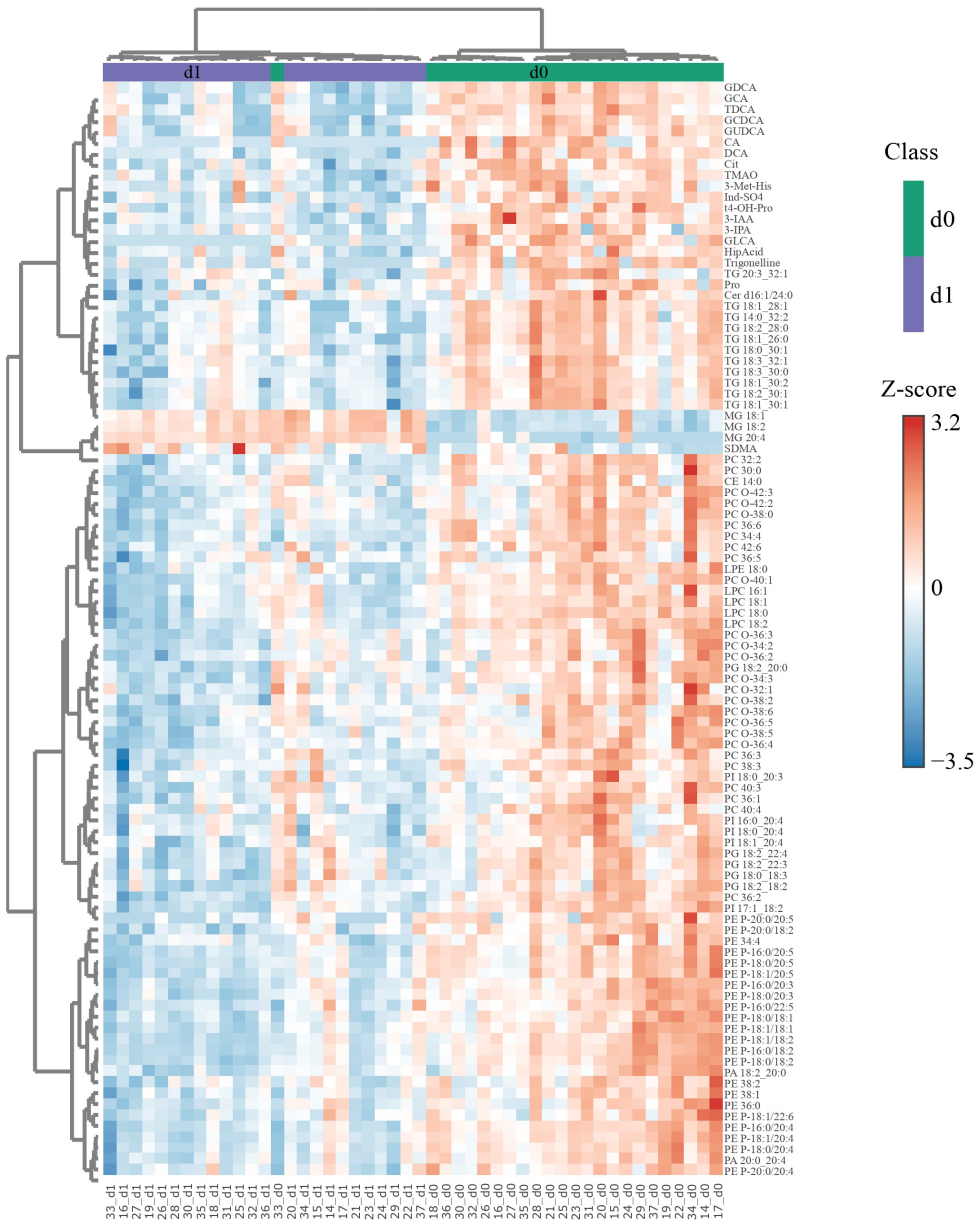
Differences in metabolite concentrations distinguish nearly perfectly between d0 and d1 samples. The 100 most significant (FDR obtained with *t*-test = 1.3e^−9^-5.86e^−5^) differentially abundant metabolites were selected by Euclidean distance for d1 with respect to d0 and subjected to unsupervised hierarchical biclustering analysis. Each colored cell in the heatmap corresponds to the group average concentration of the analyte with respect to the mean-centered and divided by standard deviation of the analyte (*z*-score). *Y*-axis = metabolite dendrogram, *n* = 24 (d0) and 24 (d1). The respective analyses for d3 vs. d0 and d7 vs. d0 are shown in Supplementary Figures S2a, b, respectively.

A hierarchical clustering analysis based on the 100 most differentially abundant metabolites revealed a high degree of separation between the pre- and post-operative samples at all three time points, whereby all postoperative samples clustered in their own clade only on d1 (Figure 2, Supplementary Figure S3). There was an overall tendency toward lower analyte concentrations on d1, but MGs constituted a notable exception in that concentrations of the three MG among these 100 analytes were higher post-op. Furthermore, this analysis revealed a strong coregulation of metabolites within one class, es exemplified by the clades 1–4, marked by a red circle, containing mostly BA (clade 1), TG (clade 2), PC/LPC (clade 3), and PE (clade 4). Co-regulation within metabolite classes (particularly within PS, PI, PG, PE, PA, TG, LPC, CE, BA, and AA) is also evident in a matrix based on correlations among all metabolites (Supplementary Figure S4). This analysis also revealed between-class correlations, for instance of PI with PG and PA and of TG with PC, a subgroup of LPC, and DG.

### Two distinct kinetic patterns emerge from plasma metabolite abundance analysis

**Figure 3:**
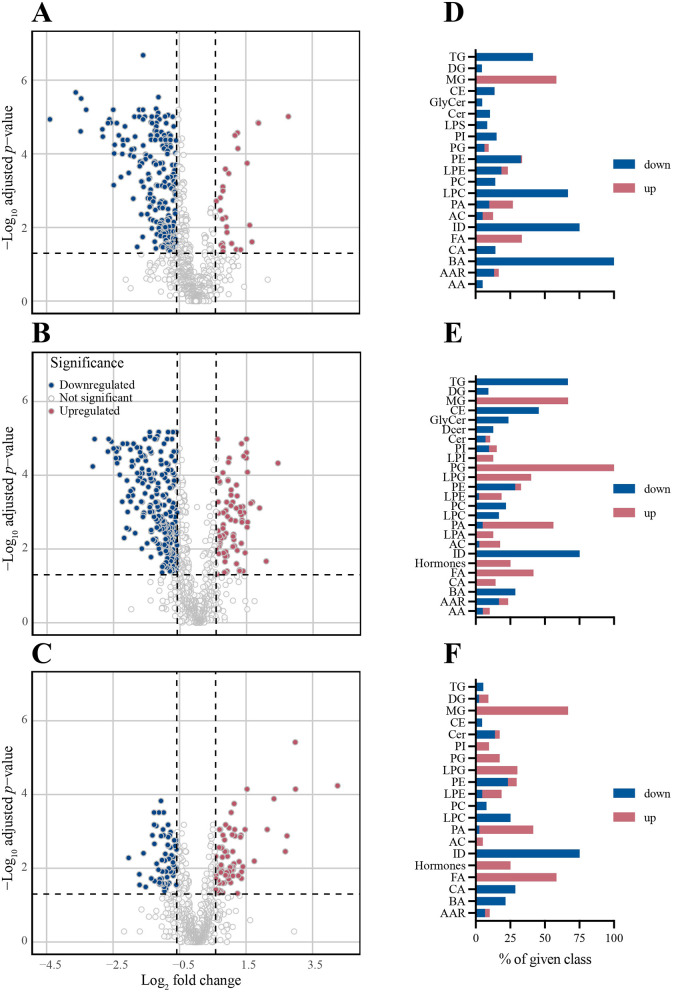
Differential abundance analysis based on the same metabolites as Figure 1 reveals a shift from down- to upregulation during post-operative recovery. Differentially abundant (FC ≥|1.5|, FDR < 0.05 Wilcoxon rank-sum test) metabolites with respect to d0 are indicated for each time point post-op. For each day, a volcano plot indicating differential abundance of individual metabolites and a bar chart indicating the direction of abundance change in each metabolite class are shown. Blue bars = downregulation; red bars = upregulation. **(A, D)** d1. **(B, E)** d3. **(C, F)** d7 and *n* = 24 (d0); 24 (d1); 24 (d3); 20 (d7).

Results of a differential abundance (FC ≥|1.5|, FDR < 0.05) analysis are visualized in Figure 3 and summarized in Table 3. As seen in the volcano plots (Figures 3A–C) and Supplementary Figure S5 there was a trend toward higher *p* values and FC (log10) values from d1 to d7. The highest number of differentially abundant metabolites was observed on d3, but even on d7 there were still 154 differentially abundant metabolites. Overall, most of the differentially abundant metabolites were downregulated. However, the number of downregulated metabolites decreased throughout the time course, whereas the number of upregulated metabolites increased, which resulted in a slight preponderance of upregulated vs. downregulated metabolites by d7 (Table 3).

**Table 3:** Number (%) of differentially abundant metabolites post-op.

**Day post-op**	**Differentially abundant metabolites**		
	**Total no**.	**No. (%) up**	**No. (%) down**
d1	262	34 (13%)	228 (87%)
d3	334	82 (25%)	252 (75%)
d7	154	82 (53%)	72 (47%)

**Figure 4:**
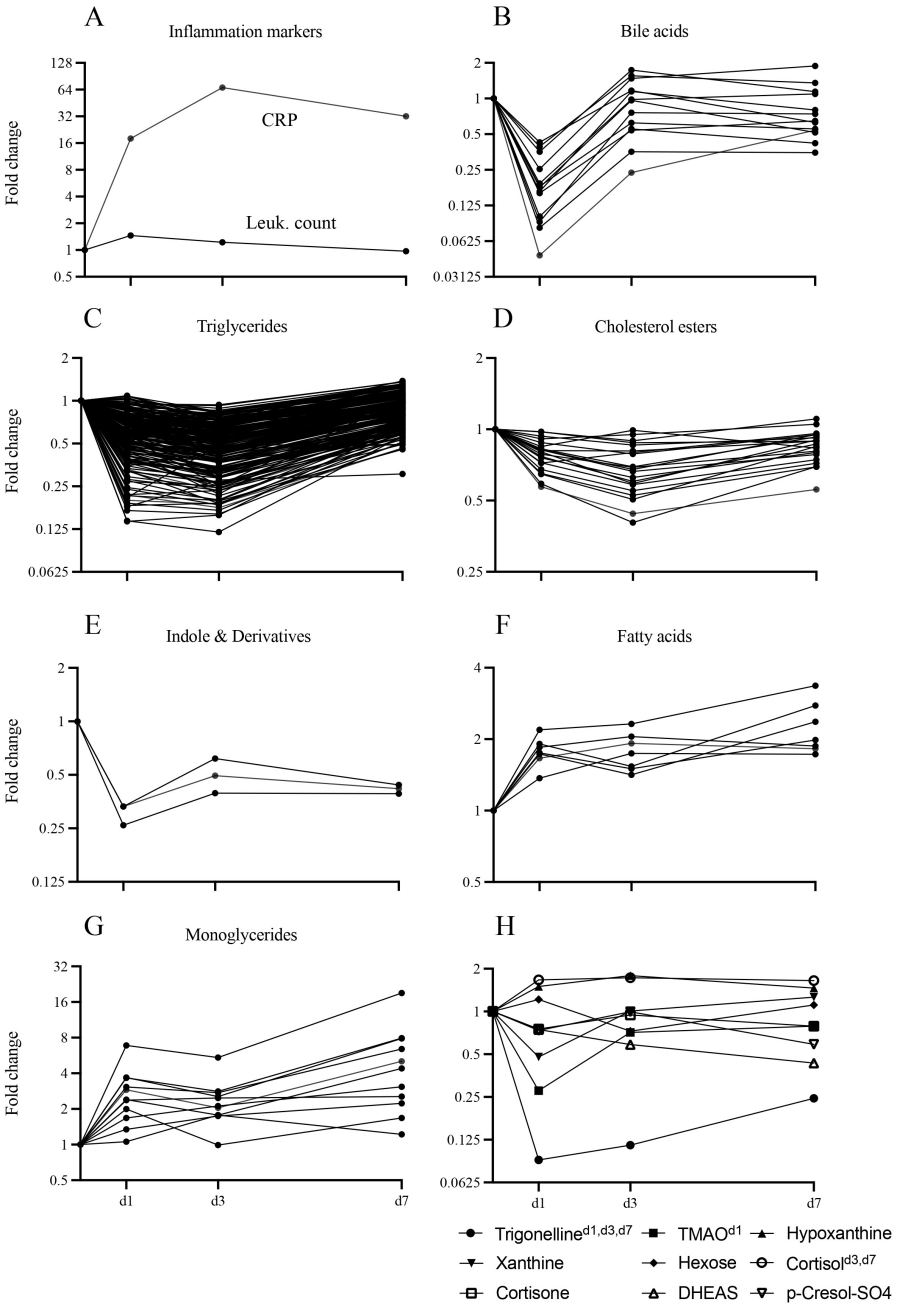
Kinetics of concentration changes in selected metabolite classes and blood inflammatory parameters throughout the time course. Concentration dynamics are expressed as fold change with respect to d0. The y-axis label “Fold change” also applies to panels **(B, D, F, H)**. **(A)** Blood CRP and leukocyte count. **(B–H)** Plasma metabolites. **(B–E)** Selected predominantly downregulated metabolite classes (bile acids *n* = 13, triglycerides *n* = 238, cholesterol esters *n* = 22, indoles and derivatives *n* = 3). **(F, G)** Selected predominantly upregulated metabolite classes (fatty acids *n* = 8, monoglycerides *n* = 11). **(H)** Selected individual metabolites of interest [cortisol, cortisone, dehydroepiandrosterone sulfate (DHEAS), hexoses, hypoxanthine, p-cresol-SO4, trigonelline, trimethylamine N-oxide (TMAO)]. Days on which fold change was significant with respect to d0 are indicated as superscripts. *n* = 24 (d0); 24 (d1); 24 (d3); 20 (d7).

Metabolite classes could be grouped into two kinetic patterns: those that were consistently downregulated (TG, BA, CE, ID) and those that were consistently upregulated (FA and MG; Figures 3D–F, 4, Supplementary Figures S6a, b). Notably, all BA were downregulated on d1 and 35.7% remained downregulated more than 1.5-fold on d3 and d7 (Figure 4B). Likewise, LPC (Supplementary Figure S6a) and ID (Figure 4E) were consistently downregulated. Downregulation of TG was most pronounced on d3 (66%), but nearly normalized by d7 (Figure 4C). Substantial persistent upregulation was observed with FA and MG (Figures 4F, G). Of the metabolite groups with few or only a single member, substantial downregulation of three metabolites was noted (Figure 4H). (i) Concentrations of the alkaloid trigenolline were very low on d1 and reached only about 25% of pre-op levels by d7. Trigenolline is found in foods, notably in coffee beans, where it makes up about 1% and can also originate from gut microflora (27). (ii) Similar downregulation was observed with trimethylamine N-oxide (TMAO), which can originate from gut microbiota or hepatic metabolism of trimethylamine. TMAO is closely related to the so called “western diet,” which is rich in choline and carnitine, and is associated with cardiovascular disease (28). (iii) 3-IAA is a Trp metabolite which is a common growth hormone in plants, but also an intermediate of Trp catabolism in humans. Thus, the initially low, but then rising plasma levels of trigenolline and TMAO likely reflect absence of oral alimentation on d1, and the gradual resumption of an oral diet thereafter. There was a mild-to-moderate increase in cortisol and consecutive decrease in dehydroepiandrosterone sulfate (DHEAS) post-operatively (indicating a physiologic stress response), whereas cortisone levels did not increase.

### Correlations between metabolites and clinical parameters reflects strong systemic inflammation

**Figure 5:**
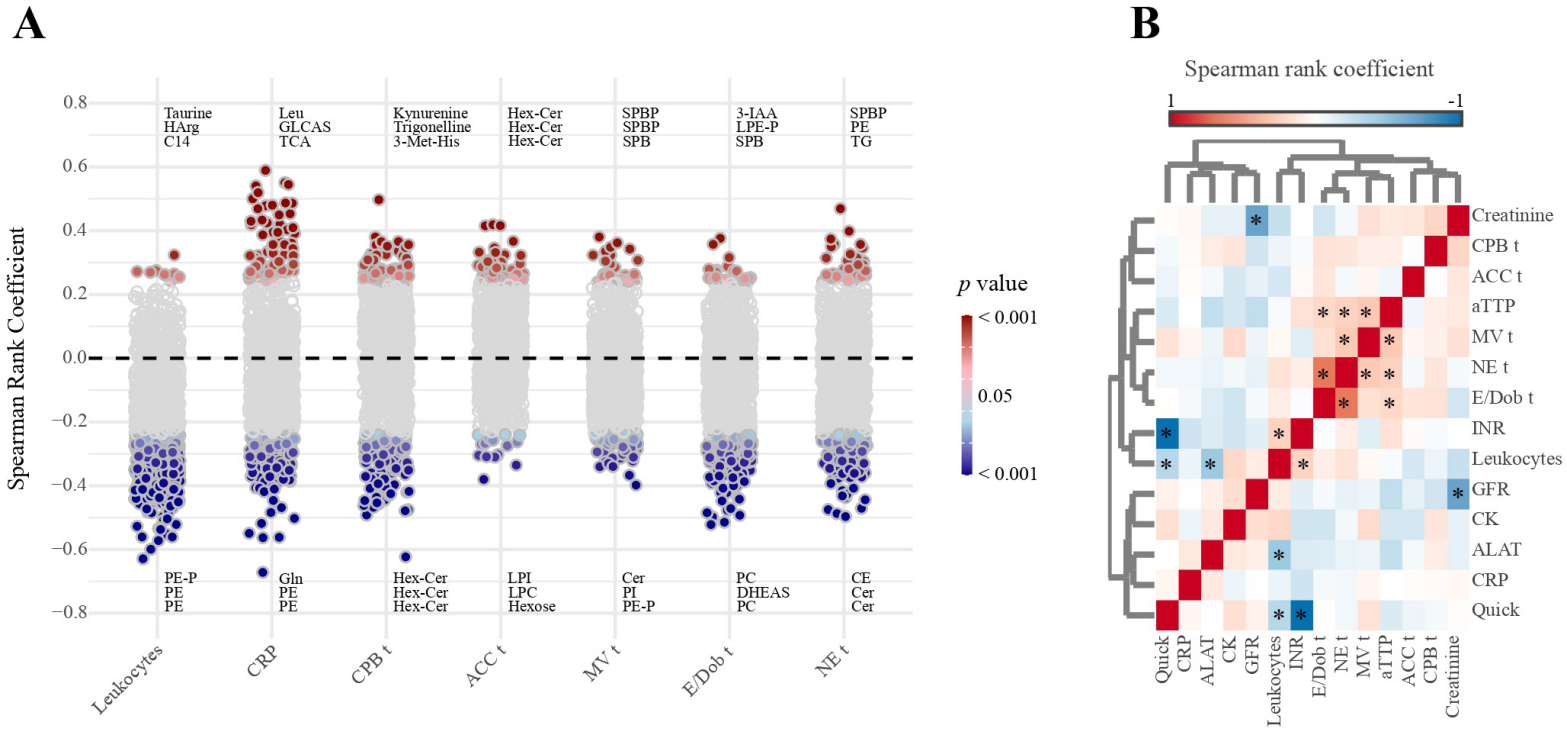
Correlations between intra-operative parameters, inflammation markers, and metabolites, based on Spearman rank coefficients. **(A)** Correlation with metabolites, pooled analysis of data from all three time points combined. Each circle indicates one metabolite. Labeled metabolites represent the ones with highest correlation coefficient. Gray fill color = *p* > 0.05. **(B)** Correlations among intra-operative parameters (*n* = 5), inflammation markers (*n* = 2), and clinical chemistry parameters (*n* = 6). * = *p* < 0.05. *p* values based on FDR obtained with *t*-test. *n* = 24 (d0); 24 (d1); 24 (d3); 20 (d7).

We then aimed to identify clinical parameters that are most closely associated with changes in metabolite abundance and can be considered the main drivers of the marked metabolic alterations post-op. Among the intra-operative variables, the most significant correlations with metabolites at all three time points were observed for NE time, CPB time, and E/Dob time (Figure 5A, Supplementary Figure S8, Table 4). However, most significant correlation coefficients were of only modest strength, ranging between 0.25–0.5. On the other hand, substantially stronger correlations were observed between the markers of inflammation (CRP and leucocyte count) and metabolites, indicating that systemic inflammation is a strong, but certainly not the only, driver of the observed metabolite alterations. We then tested whether we could identify intra-operative parameters responsible for the observed systemic inflammation. However, the only significant correlation with inflammation was between ALAT and leukocytes (*r*= −0.41, *p* = 0.0006; Figure 5B). Furthermore, Spearman rank correlation analysis revealed a significant association between ICU stay and a single metabolite (PI 16:1_18:2; ρ = −0.72, FDR = 0.033) on postoperative day 3. No consistent associations were found between metabolites and ICU stay at other time points (data not shown).

**Table 4:** Number of significant correlations (*p* ≤ 0.05) between metabolites and clinical parameters.

**Parameter^a^**	**d1**	**d3**	**d7**	**Sum**
CRP	152	32	54	238
Leukocyte count	66	123	29	218
E/Dob time	72	63	46	181
NE time	85	37	46	168
CPB time	56	64	47	167
MV time	53	21	27	101
ACC time	34	20	27	81
All	518	360	276	1,154

^a^Arranged in descending order according to sum of significant correlations.

CRP, C-reactive protein; E, epinephrine; Dob, dobutamine; NE, norepinephrine; CPB, cardiopulmonary bypass; MV, mechanical ventilation; ACC, aortic cross clamping.

## Discussion

The use of targeted metabolomics to profile longitudinal plasma metabolite changes in CAD patients undergoing CABG fills an important gap in the literature as our study is the first to show dynamic alterations of plasma metabolite changes across four clinically meaningful timepoints, spanning both acute and recovery phase after CABG, offering new insight into the trajectory of metabolic recovery and the influence of surgical and inflammatory stress. This distinguishing it from similar studies (29, 30), as most prior studies have either focused on static metabolic profiles in chronic CAD or have lacked a time-resolved approach. Although changes in metabolites were evident during the acute phase and normalized during the recovery phase, intriguingly, there were few metabolite classes, belonging to the lipid, bile acid, and Trp derivatives, which exhibited a steady kinetic pattern throughout our investigations. Notably, plasma TG levels were significantly downregulated and were associated with increases in both MG and FA, suggesting perioperative modulation in lipid metabolism. Such alterations in metabolites could occur due to several plausible factors, including surgical trauma-associated inflammatory milieu, fasting, catecholamine-induced lipolysis, and heparin treatment. Of note, disturbances in gut microbiota community also play a significant role in inducing changes in metabolite profiles (31).

Increased systemic inflammation is a well-known consequence of CABG, in the process of which macrophages with inflammatory^high^ phenotype tend to accumulate plasma-circulating TG, by either endocytosis or receptor-mediated internalization (32). Such TG-laden inflammatory macrophages exhibit reduced capacity for lipolysis, which further lowers the inflammatory attributes of macrophages associated with increased mitochondrial dysfunction, decreased ability to polarize, migrate (33), and phagocytose, and reduced prostaglandin E2 production (34). This suggests a negative feedback loop to minimize the inflammatory state of these macrophages upon intracellular TG accumulation and could explain the reduced post-operative plasma TG levels. On the other hand, TG, CE, and PC accumulate in intracellular lipid droplets as cells respond to stress (34–36). In our cohort, surgery-induced acute stress could promote lipid droplet turnover to compensate the energy and redox imbalances, where TG are constantly being hydrolyzed (via lipolysis or lipophagy) to convert into free FA and MG (37), as reflected by their consistent upregulation in postoperative CAD circulation. Of note, CE-mediated lipid droplet nucleation is facilitated by these intracellular accumulated TG, since reduced plasma CE were also evident in our cohort (38). Our findings align with those of Ding et al. (39) who reported a significant reduction in specific cholesterol ester species (CE18:0, CE16:0) in patients developing sepsis following cardiac surgery with CPB. Their lipidomic analysis aimed to characterize the molecular signature of sepsis in this context. However, interpretation is limited by the use of sequential organ failure assessment (SOFA) ≥2 as a sepsis criterion, which may not reliably distinguish infection from postoperative inflammation, and by the heterogeneity of the cohort, including prolonged CPB durations and ICU stays (39). Despite these limitations, the lipidomic alterations reported by Su et al. are consistent with our data and support the notion that CABG-associated inflammation and physiological stress drive shifts in plasma CE levels, contributing to disrupted postoperative lipid homeostasis. Another contributing factor is the state of fasting, where the entire patient cohort remained fasting ≥6 h prior to surgery until an oral diet was resumed on the first postoperative day. In addition to cardiac surgery stress (40), this nutrient stress also triggers the sympathetic nervous system (41) to secrete catecholamines (norepinephrine) that activate lipolysis to satisfy the cellular energy demand, by promoting the breakdown of stored TG into FA and MG.

Furthermore, every patient received high-dose intravenous (IV) unfractionated heparin (400 IU/kg body weight) before commencement of extracorporeal circulation, followed by low-dose prophylactic therapy until discharge. Since IV administered heparin rapidly increases lipid hydrolyzing enzymes (lipoprotein lipase), there could be a massive hydrolysis of TG into FA and MG (42). Likewise, the entire patient cohort were uniformly treated with opioids, as part of peri- and post-operative pain management medications. Opioid could be regarded as a significant driving factor for changing cellular metabolism (43). In this context it is plausible that opioid administration may contribute to altered lipid metabolism, promoting lipolytic activity. This hypothetical mechanism could, in part, facilitate the mobilization of FA (44) and MG from intracellular TG stores in response to increased cellular energy demands (45).

PC, as predominant class of plasma lipids, was consistently decreased in circulation, possibly due to their role in lipid droplet biogenesis (46) for high-energy supply during surgery-induced inflammation. Another explanation could be PC uptake via the CD36 scavenger receptor, increasing the cellular PC pool in differentiating macrophages, which may accelerate PC turnover and amplify inflammation (47). Additionally, monocytes, as macrophage precursors, feature a higher intracellular PC content, particularly with aging (48). These concepts hold true in our cohort settings that are representative of an aged population together with ongoing post-surgical inflammation. We therefore propose that an increased demand for intracellular PC content might reflect decreased circulating PC in the blood stream. A study of plasma lipid changes in community-acquired pneumonia (CAP) reported lower PC concentrations during acute presentation, which normalized with clinical recovery (49). These results agree well with ours, as acute CAP features pronounced systemic inflammation of a similar magnitude as our cohort. Interestingly, a reduced plasma PC/PE ratio has led to increased levels of NLRP3, an integral component of the inflammasome activation pathway, which promoted changes in left ventricle geometry among CAD

patients with insulin resistance (50). Since 50% of our CAD patients have diabetes as comorbidity, a notable decrease in plasma PC/PE ratio could possibly pose an increased risk to these patients in terms of cardiac remodeling. Similar to our findings, plasma levels of PC were also reduced in patients with other chronic inflammatory diseases, such as Alzheimer's disease (51) and diabetic nephropathy (52), where both plasma PC and PC-Os were inversely associated with renal-related clinical covariates [estimated glomerular filtration rate (eGFR) and Albuminuria], end-stage renal disease (ESRD) and all-cause mortality in diabetic nephropathy (52). Sometimes, choline-deficient diet could also explain the reasons behind a sudden drop in plasma PC levels (53, 54) among CAD patients. However, a significant decline in plasma lipids could explain an ongoing catabolism due to acute stress (55), induced by surgical trauma. Further, these circulating PC might have sequestered by binding to structurally-altered CRP (56) due to massive inflammation. Since PC is further cleaved by phospholipase A2 activity to generate LPC (8), we noticed substantially reduced plasma LPC in CAD patients, which could reflect a limited availability of its precursor molecule, PC, in the circulation. Similar to our findings, plasma LPC levels were decreased among patients with obesity and diabetes (57) as well as Alzheimer's patients with chronic inflammation (58). Since LPC act as a carrier molecule for polyunsaturated fatty acids (PUFAs) (58), a deficit of plasma LPC pool might limit supply of PUFA to vascular health, causing vascular dysfunction (59), increased inflammation and all-cause mortality (60).

Several reports have shown alterations in gut microbiota composition following cardiac surgery together with CPB usage (61, 62). Such gut dysbiosis led to decreased LPC generation which was, in particular, associated with reduced colonization of *Bacteriodes* population, as investigated in chronic pathologies such as Alzheimer's disease (63). Furthermore, we speculate that there was increased ferroptosis with decreased plasma LPC (63) in our patient cohort, as iron accumulation and lipid peroxidation have been reported following cardiac surgical procedures, including the usage of CPB (64, 65). However, the notion that ferroptosis directly mediates LPC depletion remains hypothetical and warrants further investigation in the context of our CAD cohort. In line with the above-mentioned beneficial role of PC and LPC, our study shows a negative correlation between PC and ether-linked phosphatidylcholine (PC-Os) as well as LPC and acute inflammatory markers (CRP and leucocyte count) and the use of CPB during surgery, highlighting a possible role of PC and LPC in regulating ongoing inflammation.

Changes in plasma bile acids were also noticed in our CAD cohort, where BA metabolites were consistently downregulated, especially on postoperative day 1 and 3. This could reflect limited generation of BA metabolites due to gut dysbiosis (66), which was also evident in patients with other chronic inflammation like ulcerative colitis (66). Hence, to this extent, there is a high likelihood for a surgical trauma-induced gut dysbiosis in decreasing circulating BA metabolites postoperatively CAD. In fact, increased BA concentrations in the circulation have been reported to modulate blood monocyte function upon specific activation of bile acid receptor (TGR5). These monocytes exhibit decreased phagocytic capacity and reduced production of pro-inflammatory cytokines (67) during bacterial infection. Nevertheless, bile receptor TGR5 agonism induces nitric oxide (NO) production and hampers the adhesion of circulating monocyte to vascular endothelium (67). In line with these reports, we supposed whether bile acid synthesis was reduced or their bioavailability was diminished due to their consumption at cell-expressed receptor binding sites in our cohort. But, in our previous study (68), we demonstrated IL-6^high^ producing blood mononuclear cells among postoperative CAD patients, explaining the existence of functional monocytes and therefore, the possibility for decreased BA synthesis. Thus, a deficit in bile acid production could pose an increased risk for amplified inflammation in our postoperative cohort. Intriguingly, BA was positively correlated with CRP in these patients, suggesting a counteracting role of bile acids to reduce ongoing inflammation. This notion was supported by a report showing that increased CRP levels in lipopolysaccharide-induced endotoxemia among healthy volunteers correlate with a rise in total BA concentration (69) where these increased circulating BA metabolites exhibited immunosuppressive properties. However, a functional relationship between CRP and circulating BA would be interesting to investigate in our CAD cohort. Interestingly, a study on the effect of fasting and refeeding on plasma BA metabolites in mice demonstrated that both primary and secondary BA were decreased during fasting and this condition was reversed upon refeeding. These alterations were largely associated with decreased abundance of BA-metabolizing *Lactobacillus* and *Bifidobacterium* in gut. In addition, the genes involved in hepatic BA synthesis such as cholesterol 7 alpha-hydroxylase (CYP7A1), Oxysterol 7 alpha-hydroxylase (CYP7B1), and aldo-keto reductase family 1 member d 1 (AKR1D1) were repressed during the fasting state. However, BA metabolizing microbiotas as well as hepatic BA synthesizing genes were expressed during re-feeding (70). A similar mechanism might underlie the reduced plasma BA levels in our CAD patients, who were also in the state of fasting and gradual refeeding during the peri-and post-operative period. Of note, multiomic study involving metabolomics as well as metagenomics.

Next, a consistent downregulated pattern of indole derivatives (indole, 3-IAA, 3-IPA and Ind-SO_4_) was noticed. These molecules are generated as Trp catabolites by the gut microbial community (22), are absorbed by intestinal epithelium and enter the blood stream. They are particularly known to fortify intestinal epithelial barrier function, by increasing the expression of tight junctional proteins, and thereby to prevent leaky gut (22). Post-surgical gut microbial dysbiosis could explain the reason for reduced plasma indole derivatives in our cohort. To support our findings, significantly reduced levels of indole derivatives, in particular 3-IAA and 3-IPA, have been reported in patients with severe atherosclerosis (71), indicating their anti-inflammatory role (72). Additionally, 3-IPA is known to enhance systemic homeostasis with the gut-multiorgan axis during acute systemic inflammation (73). Although several indole derivatives promote inflammation-attenuating host responses, indole sulfate is considered to be the notable exception, which is a uremic toxin and accumulates in serum of chronic kidney disease patients (74). In line with this and despite reduced levels of indole sulfate in our cohort, a positive correlation between indole sulfate and CPB time was noted, reflecting its possible role in oxidative stress. In addition to this, another Trp catabolite (kynurenine) and trigonelline were positively associated with CPB time. Due to their immune suppressive qualities, both kynurenine and trigonelline could be involved in regulating the stressful inflammatory milieu during cardiac surgery. However, specific functions of these metabolites should be investigated. Besides, growing evidence suggests that gut microbiota can translocate following intestinal ischemia-reperfusion injury during surgery, leading to disruptions in host microbial ecology. To explore this, a study protocol has been developed to investigate microbial and metabolic profiles in patients with CAD undergoing CABG, aiming to understand fever of unknown origin and to improve sepsis stratification (75). This integrative metagenomic and metabolomic approach seeks to clarify how gut microbial shifts drive systemic metabolic changes and postoperative fever (75). This further highlights that reduced microbial diversity and their translocation may increase sepsis risk in this CAD population.

Lastly, the observation that inflammatory markers correlated more strongly with metabolite changes than intraoperative parameters supports the notion that systemic inflammation, rather than the surgical procedure itself, is the primary driver of metabolic disruption, which has potential implications for anti-inflammatory strategies in postoperative care. Henceforth, this study reveals time-resolved post-surgical changes in plasma metabolites that are tightly associated with surgical stress, inflammation, and gut dysbiosis, among CAD patients. Decreased PC, LPC, and CE, alongside increased FA and MG, indicate catabolic stress, mitochondrial dysfunction, and lipolysis, possibly in patients with type 2 diabetes mellitus (T2DM) or obesity. These identified metabolite trajectories could serve as molecular signatures for identifying patients at high risk of adverse perioperative outcomes. As clinical interventions, pre-operative and early postoperative nutritional support, particularly choline-enriched diets, may attenuate the observed declines in plasma PC and LPC, supporting vascular and metabolic homeostasis (76) and potentially improving patient recovery trajectories. Furthermore, sustained decreases in indole derivatives and bile acids suggest gut barrier dysfunction and microbial dysbiosis. Targeted, time-specific administration of probiotics or synbiotics, particularly those promoting *Lactobacillus* and *Bifidobacterium*, may restore microbial-derived metabolites (BA, indoles), modulate host inflammatory responses, and support intestinal barrier integrity (77). Further development of a perioperative metabolic risk score, intergrating lipidomic and microbiota-related metabolite markers (e.g., PC, LPC, FA, BA, indole derivatives), may enable improved identification of patients needing intensive monitoring, targeted nutritional support, or anti-inflammatory therapies.

However, this study has several limitations, including a relatively small sample size and missing data for four patients, particularly on day 7, which may reduce statistical power and increase the risk of type I/II errors. Due to the lack of experimental mechanistic link for the observed metabolites, our findings should be considered hypothesis-generating rather than conclusive. Therefore, both functional as well as validation studies in a larger CAD population with similar clinical characteristics are warranted to confirm these metabolic signatures to enhance the robustness of our findings and to intergrate them into a predictive algorithms or point-of-care testing risk assessments.

## Conclusions

Taken together, our current study has demonstrated discrete changes in plasma metabolite populations during the postoperative period. Although we did not observe any distinct pattern of metabolite alterations observed with CPB usage, we presume that systemic acute inflammation, rather than surgical procedure itself, plays a significant role in aberrant regulation of metabolites. According to the observation, an excessive generation of free fatty acids in our cohort could pose an increased severity to coronary atherosclerosis (78), especially among those who are comorbid with T2DM, and obesity (79), by inducing endothelial dysfunctions and defects in insulin signaling (80) and sensitivity (81). Further PC supplementation as prophylactic measures might be an ideal option for CAD patients prior to cardiac surgery in order to compensate for reduced postoperative PC and LPC levels. A targeted investigation is needed to obtain data on gut microbial communities in CAD patients before and after surgery, especially during fasting and refeeding. A better understanding of depleted microbial taxa is essential to enrich the surgery-scheduled CAD patients with probiotics containing the target microbiota. Such microbial recolonization might benefit these patients to regulate ongoing acute inflammation by maintaining the levels of BA and indole derivatives, which were hampered in postoperative CAD patients. Hence our findings on biomarker potential of these metabolic signatures could ultimately lead to the discovery of biomarkers which will aid clinical decision making regarding perioperative care, nutritional supports strategies or individualized recovery monitoring among CAD patients after CABG surgery.

## Data Availability

The raw data supporting the conclusions of this article will be made available by the authors, without undue reservation.
