# Supplementary material for: Targeted metabolomic profiling reveals inflammation–associated longitudinal changes in plasma metabolites following on-pump coronary bypass surgery
Source: Front Med (Lausanne). 2025 Oct 30;12:1673132. doi: 10.3389/fmed.2025.1673132 (PMC12611753; doi:10.3389/fmed.2025.1673132)
Supplement: Supplementary file 1 [file Data_Sheet_1.pdf]

Supplemental Material 1 = Supplementary Figures and Tables

Targeted Metabolomic Profiling Reveals Inflammation – Associated longitudinal changes in Plasma Metabolites Following on-pump Coronary Bypass Surgery

Frieder Neu[#], Max Wacker[#], Sven Schuchardt, Sam Vargese, Georg Awad, Fakhar H. Waqas, Jens Wippermann, Frank Peßler* and Priya Veluswamy*

(#), (*): contributed equally

Contents

Figure S1……………………page 1
Figure S2……………………page 2
Figure S3a…………………..page 3
Figure S3b…………………..page 4
Figure S4……………………page 5
Figure S5……………………page 6
Figure S6a…………………..page 7
Figure S6b…………………..page 8
Figure S7..…………………..page 9

Table S1……………………..page 10
Table S2……………………..page 11
Table S3……………………..page 13

A

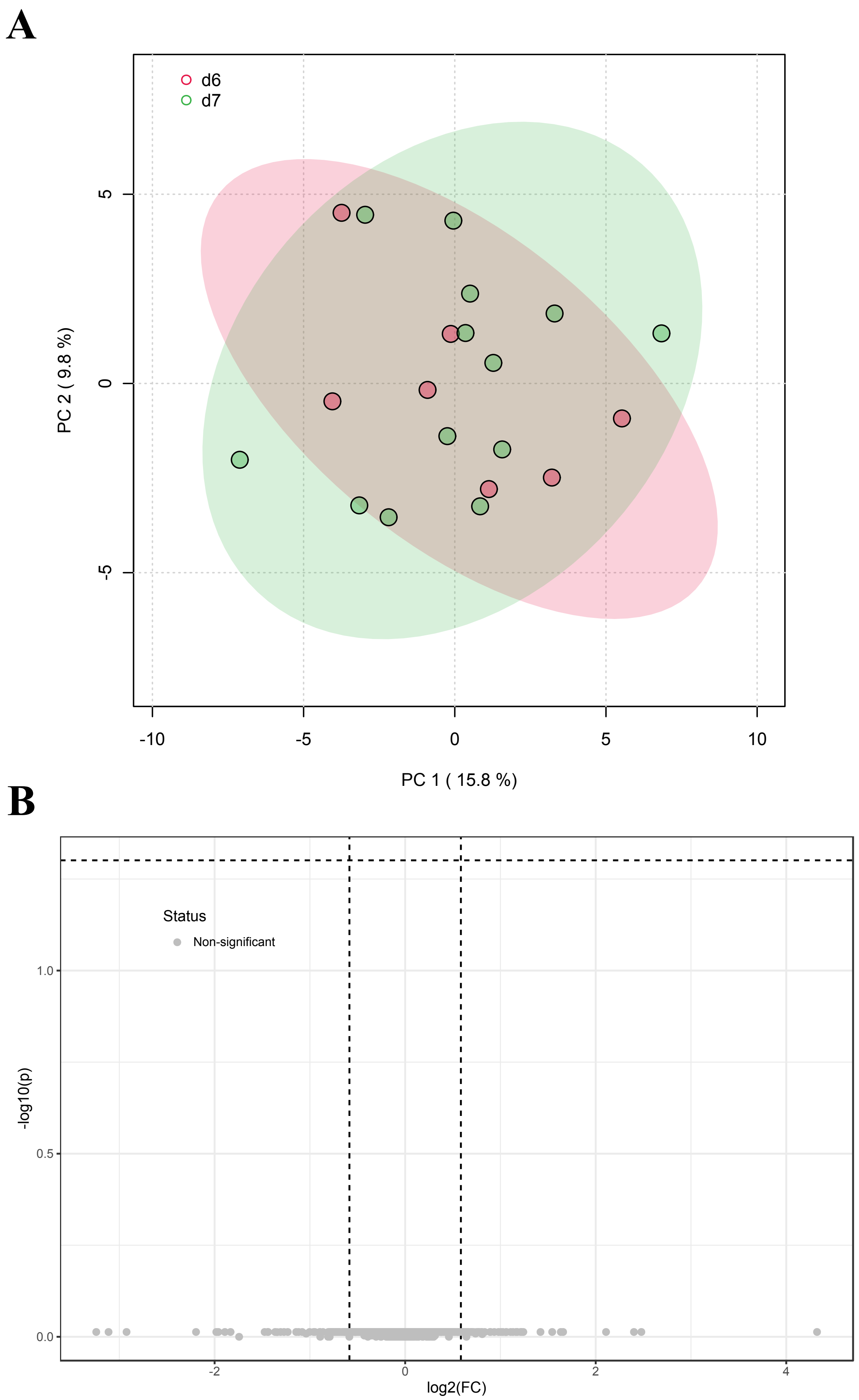

Figure S1. A two-group comparison between d6 (n=7) vs. d7 (n=13) did not reveal significant metabolic differences. Analysis is based on the same metabolites as Figure 1. **A,** Principal component analysis. **B,** Differential abundance analysis (fold change ([FC]>|1.5|, false discovery rate [FDR] < 0.05).

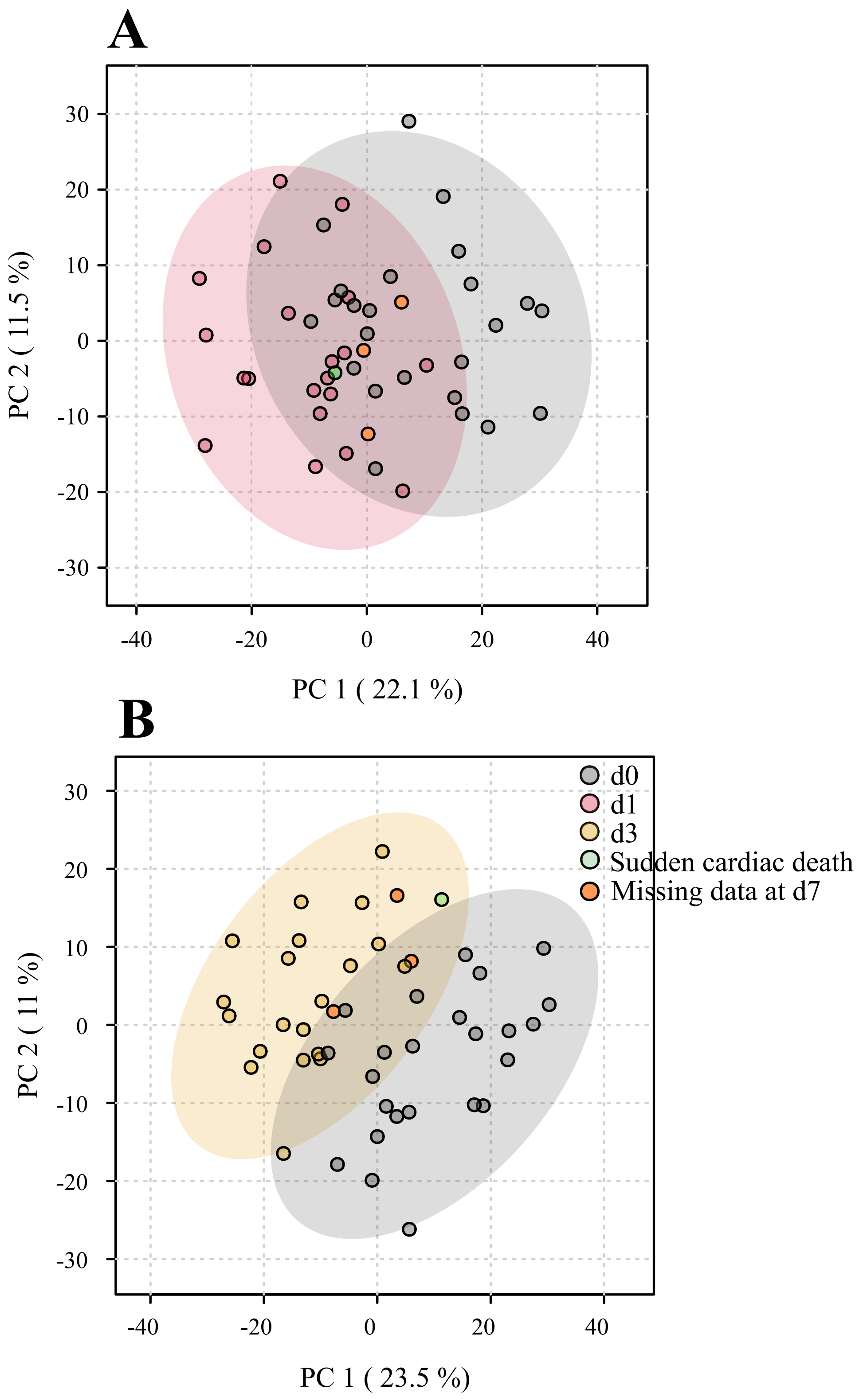

Figure S2. Metabolite patterns of the four patients with missing samples on d7 did not differ from those with a complete set of samples on d7. A principal component analysis (PCA) was performed using the same metabolites as Figure 1. **A**, d1 vs. d0. **B**, d3 vs d0.

d3 vs. d0

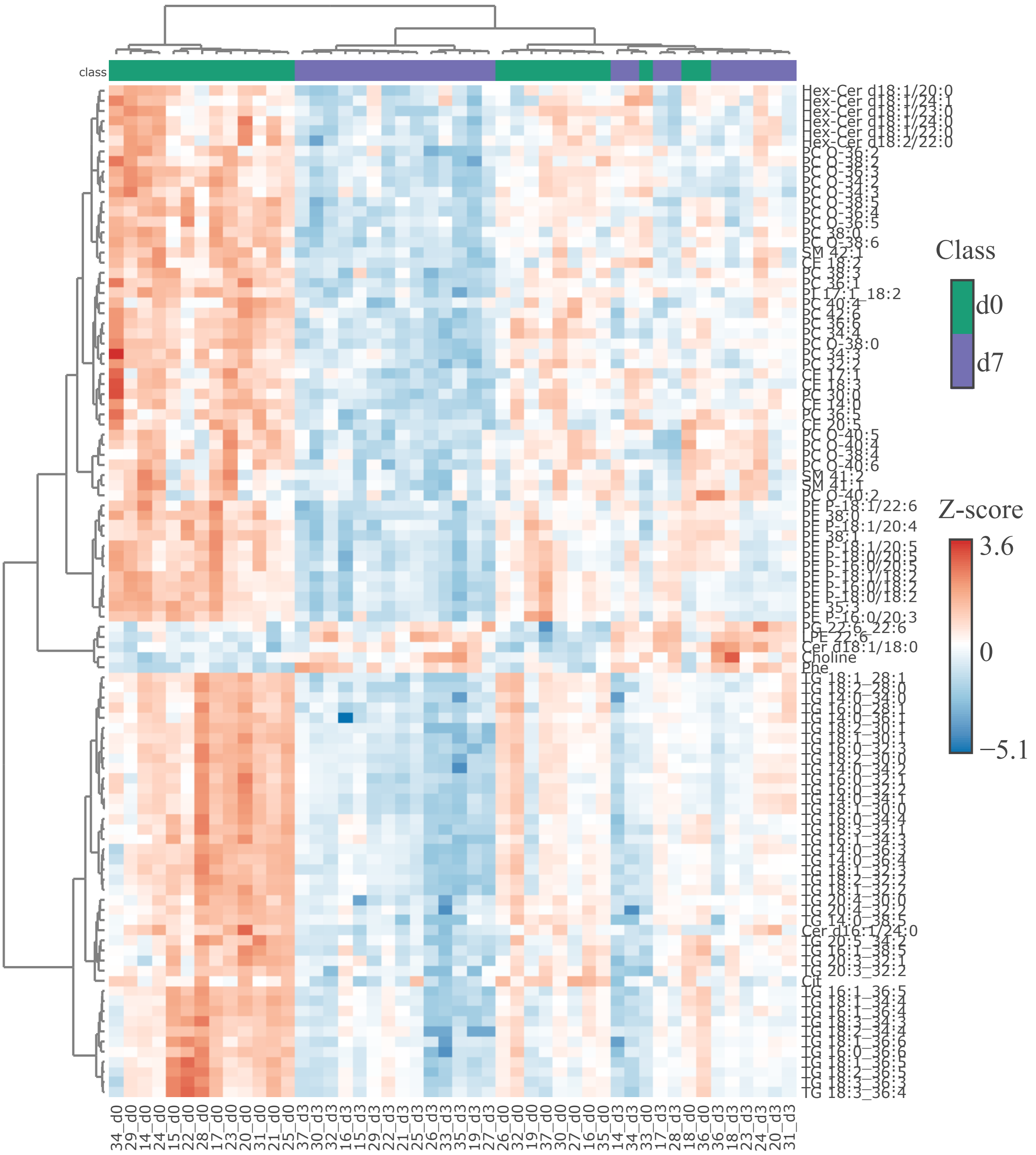

Figure S3a. 100 most significant differentially abundant metabolites selected by hierarchical biclustering analysis of d3 vs. d0 (FDR obtained with t- test 1.30e^{-9} – 5.86e^{-5}). The figure pertains to Figure 2, which shows d1 vs. d0. Each colored cell in the heatmap corresponds to the group average concentration of the analyte with respect to the mean-centered and divided by standard deviation of the analyte (z-score). Y-axis = metabolite dendrogram. *n* = 24 (d3) and 24 (d0).

d7 vs. d0

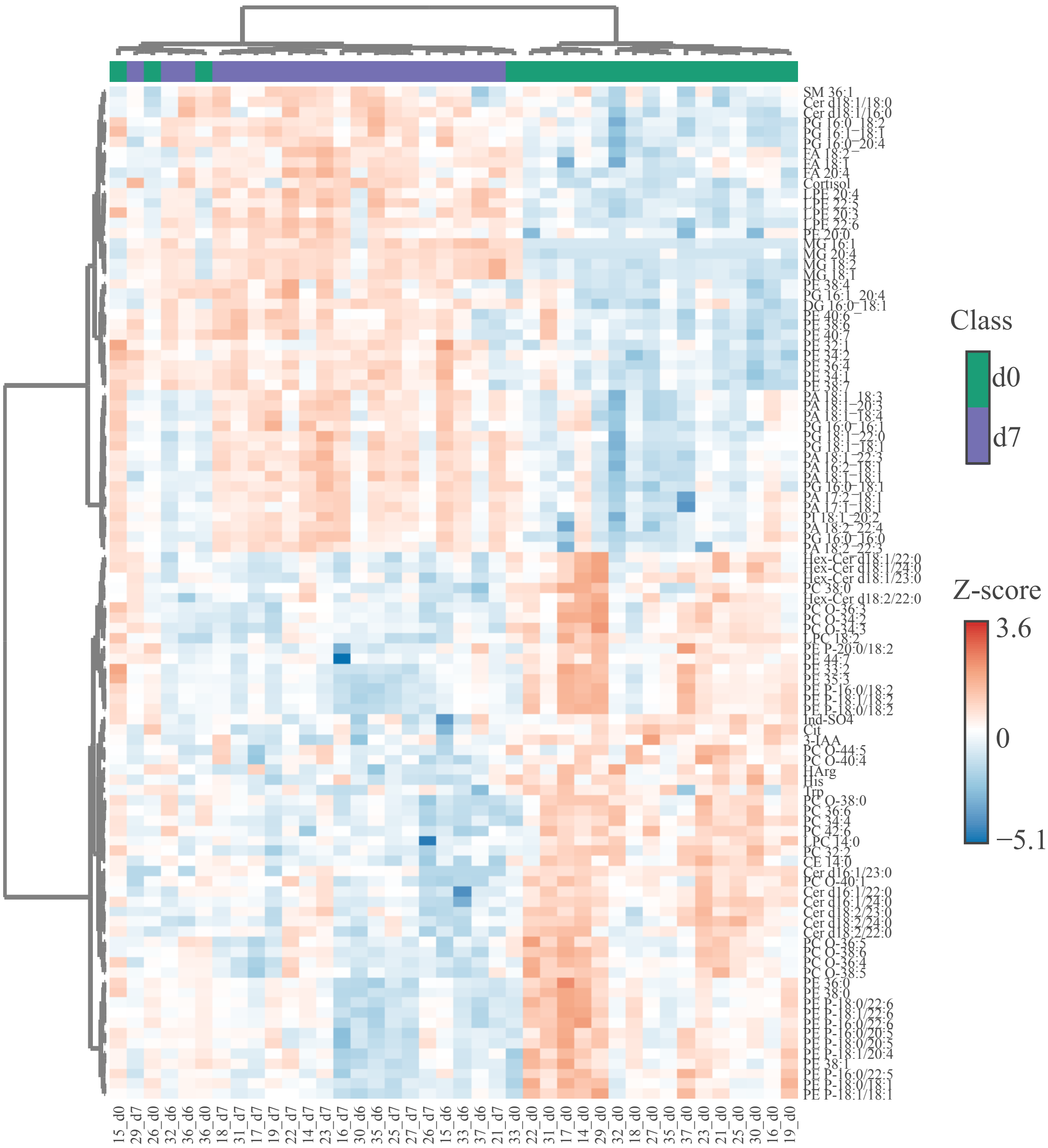

Figure S3b. 100 most significant differentially abundant metabolites selected by hierarchical biclustering analysis of d7 vs. d0 (FDR 1.47e^{-10} – 6.13e^{-4}). The figure pertains to Figure 2, which shows d1 vs. d0. Each colored cell in the heatmap corresponds to the group average concentration of the analyte with respect to the mean-centered and divided by standard deviation of the analyte (z-score). Y-axis = metabolite dendrogram. *n* = 20 (d7) and 20 (d0).

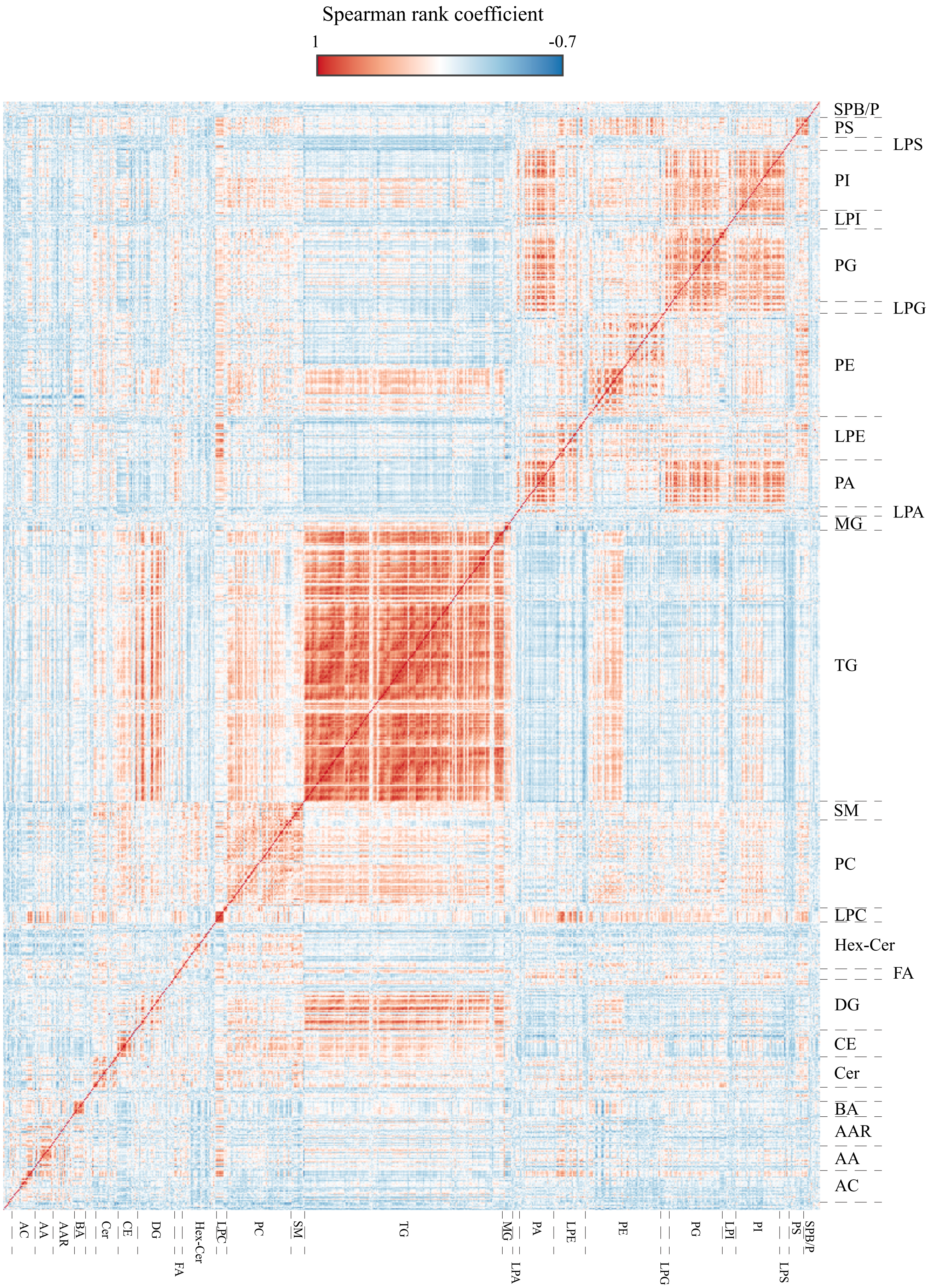

Figure S4. Correlation matrix based on Spearman rank correlation coefficients among all metabolites. Co-regulation within metabolite classes are seen. Each colored cell in the heatmap corresponds to the Spearman rank correlation coefficient of the specific metabolite. Significance was assessed by FDR obtained with t-test. Main metabolite classes are labeled on the right and below the graph. $N = 24$.

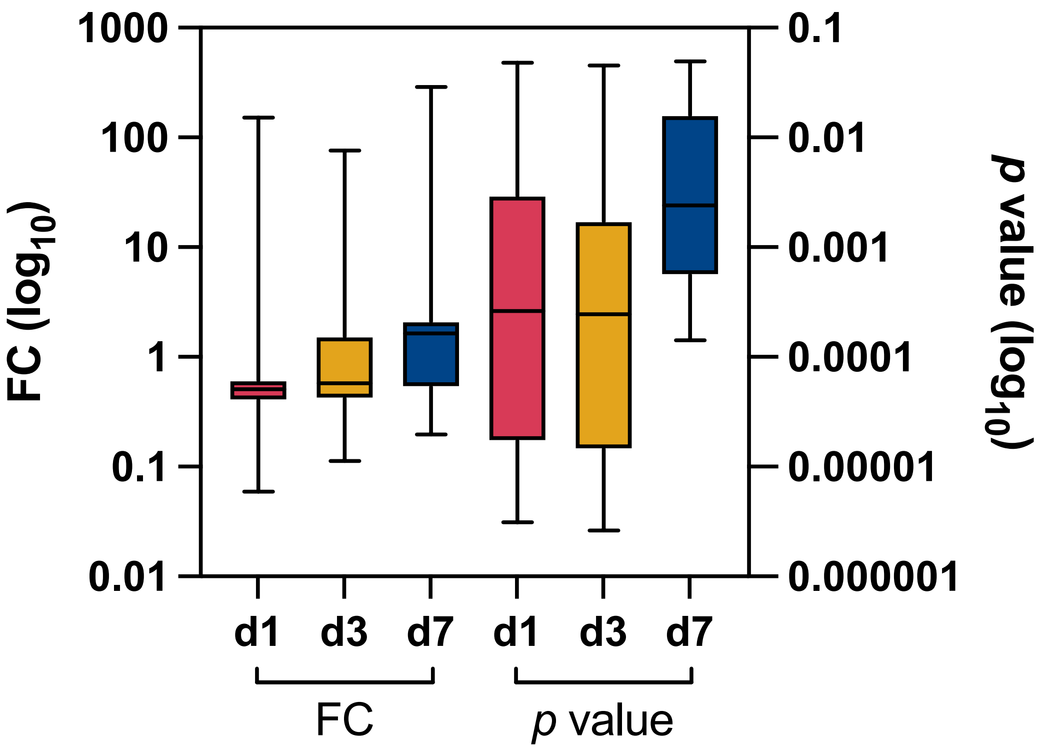

Figure S5. Fold change and p value of all metabolites at all time points postoperatively. There is a trend towards higher FC and *p* values from d1 to d7. The left y-axis represents fold change (FC) and the right y-axis the p value. $N = 24$. *P* values were calculated using FDR obtained by Wilcoxon rank-sum test.

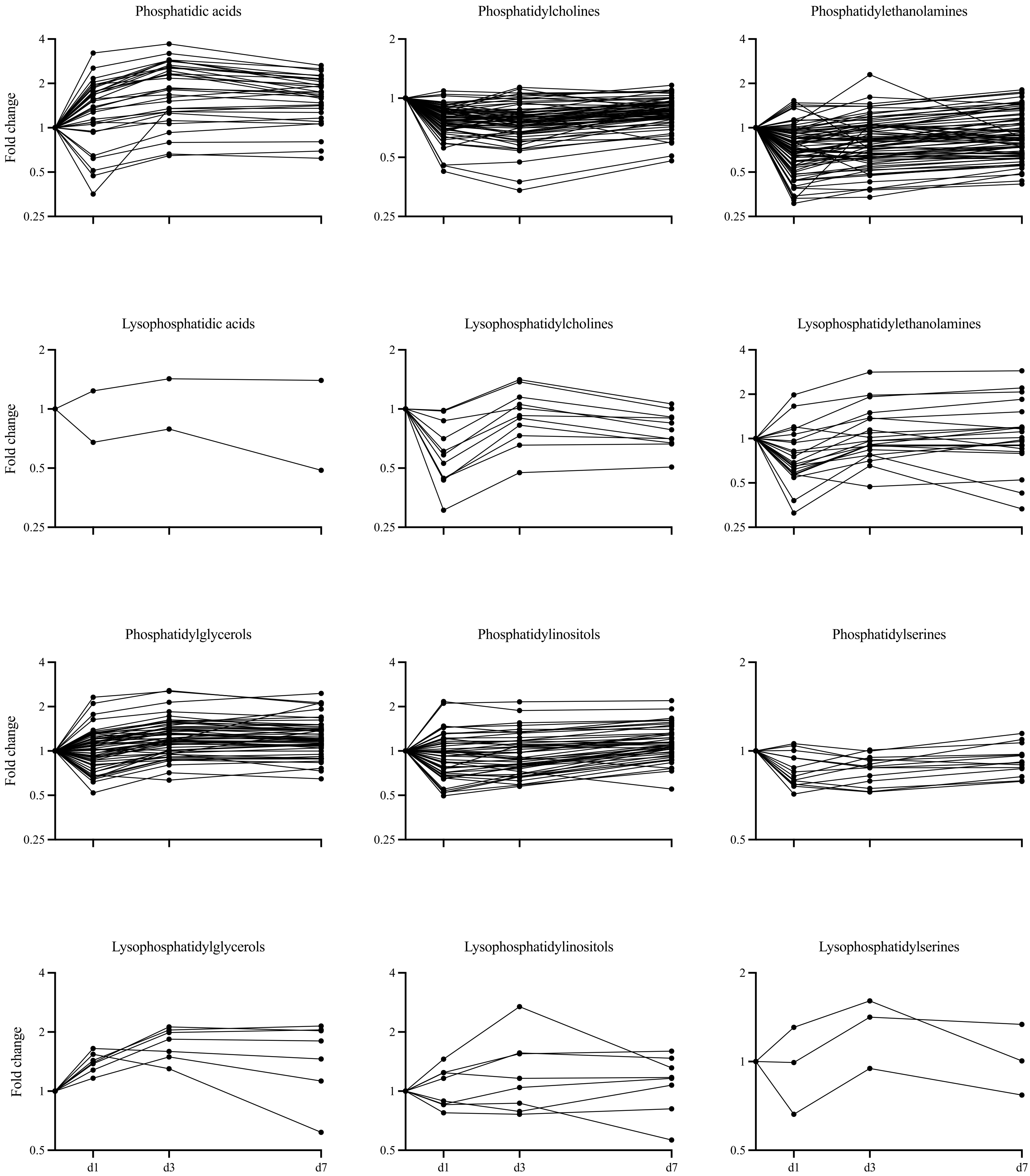

Figure S6a. Kinetics of concentration changes in the indicated metabolite classes throughout the time course. Concentration dynamics are expressed as fold change (log2 scale) with respect to d0. Corresponding class sizes are listed in Supplementary Table S1. *N* = 24

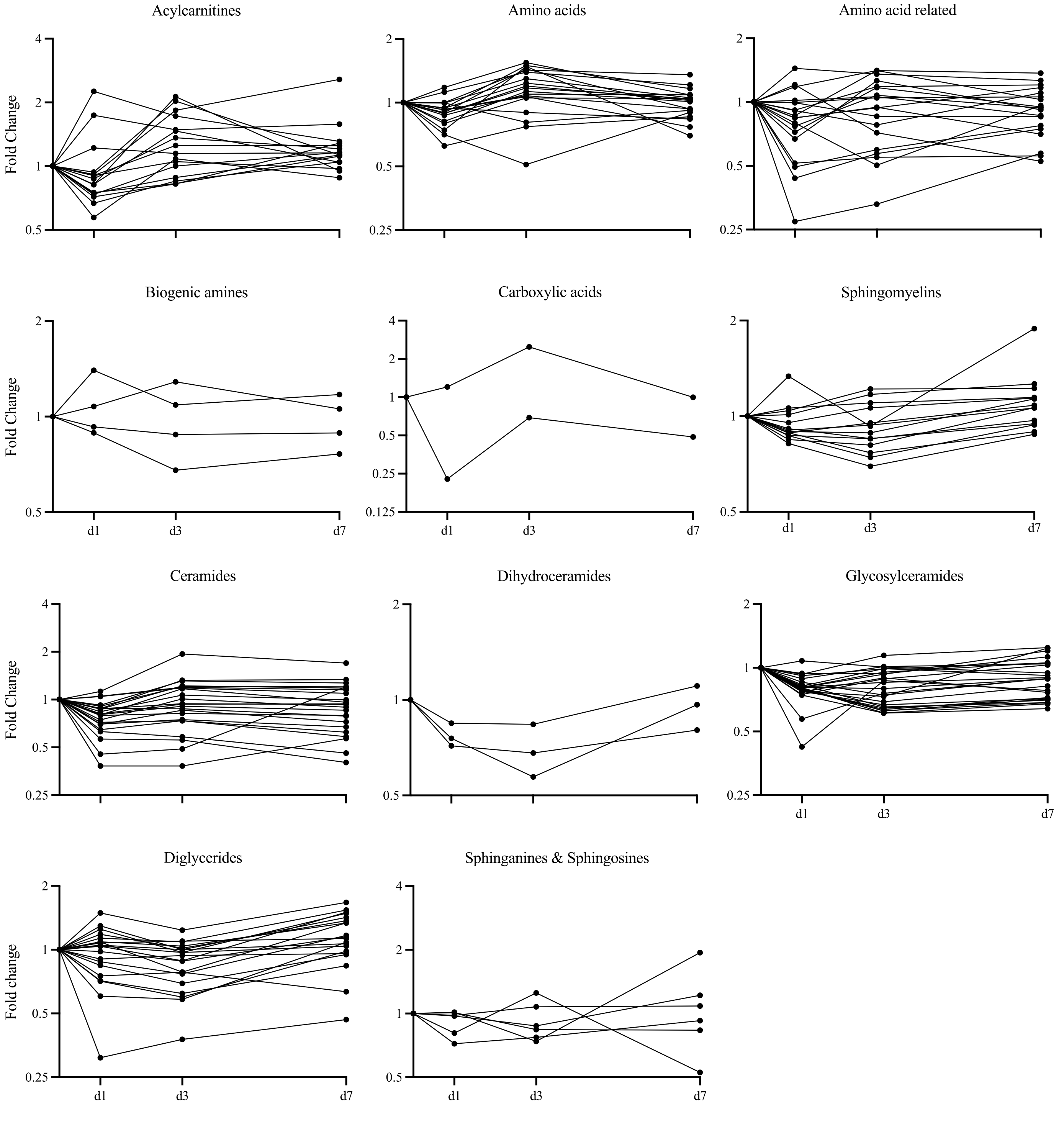

Figure S6b. Kinetics of concentration changes in the indicated metabolite classes throughout the time course. Concentration dynamics are expressed as fold change (log2 scale) with respect to d0. Corresponding class sizes are listed in Supplemantary Table S1. $N = 24$.

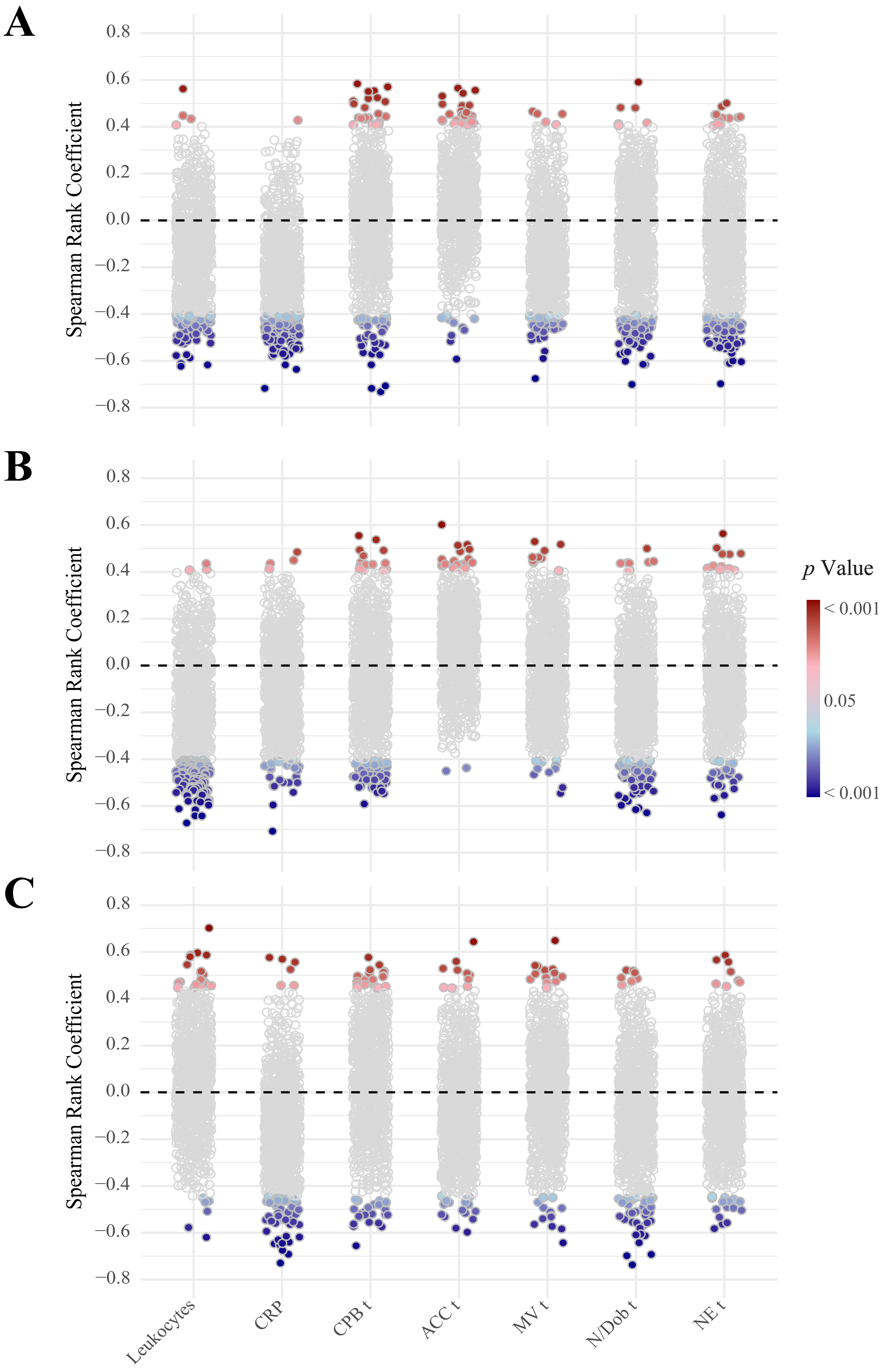

Figure S7. Correlation by day. Correlation analysis (Spearman rank coefficient) between intra-operative parameters, inflammation markers, and metabolite concentrations, separately for each time point post-op. **A**, d1 vs. d0 (n = 24), **B**, d3 vs. d0 (n = 24), **C**, d7 vs. d0 (n = 20). Each circle indicates one metabolite. Grey fill color = $p > 0.05$. P values based on FDR obtained by Wilcoxon rank-sum test.

Table S1: List of included metabolites for the three comparisons

Metabolite class	Abbreviation	d1 vs. d0	d3 vs. d0	d7 vs. d0
		**Nr. of metabolites (percent)**		
Alkaloids	Alk	1 (100)	1 (100)	1 (100)
Amine Oxides	AO	1 (100)	1 (100)	1 (100)
Aminoacids	AA	20 (100)	20 (100)	20 (100)
Aminoacids Related	AAR	29 (96.7)	29 (96.7)	29 (96.7)
Bile Acids	BA	14 (100)	14 (100)	14 (100)
Biogenic Amines	BioAm	9 (100)	9 (100)	9 (100)
Carboxylic Acids	CA	4 (57.1)	5 (71.4)	4 (57.1)
Cresols	Cresol	1 (100)	1 (100)	1 (100)
Fatty Acids	FA	8 (66.7)	9 (75)	9 (75)
Hormones	Hormones	4 (100)	4 (100)	4 (100)
Indoles Derivatives	ID	4 (100)	4 (100)	4 (100)
Nucleobases Related	NBR	2 (100)	2 (100)	2 (100)
Vitamins & Cofactors	V&C	1 (100)	1 (100)	1 (100)
Sugars	Sugars	1 (100)	1 (100)	1 (100)
Acylcarnitines	AC	26 (65)	25 (62.5)	28 (70)
Lysophosphatidic acids	LPA	8 (100)	8 (100)	8 (100)
Phosphatidic Acids	PA	41 (100)	41 (100)	41 (100)
Lysophosphatidylcholines	LPC	12 (100)	12 (100)	12 (100)
Phosphatidylcholines	PC	78 (100)	78 (100)	77 (98.7)
Lysophosphatidylethanolamines	LPE	37 (86.0)	37 (86.0)	36 (83.7)
Phosphatidylethanolamines	PE	91 (95.8)	92 (96.8)	92 (96.8)
Lysophosphatidylglycerols	LPG	10 (100)	10 (100)	10 (100)
Phosphatidylglycerols	PG	64 (100)	64 (100)	64 (100)
Lysophosphatidylinositols	LPI	16 (100)	16 (100)	16 (100)
Phosphatidylinositols	PI	53 (100)	53 (100)	53 (100)
Lysophosphatidylserines	LPS	11 (91.7)	12 (100)	11 (91.7)
Phosphatidylserines	PS	18 (100)	18 (100)	18 (100)
Sphinganines and sphingosines	SPB	8 (100)	7 (87.5)	7 (87.5)
Sphinganines and sphingosines phosphates	SPBP	8 (100)	8 (100)	7 (87.5)
Sphingomyelins	SM	15 (100)	15 (100)	15 (100)
Ceramides	Cer	27 (93.1)	27 (93.1)	27 (93.1)
Dihydroceramides	DCer	6 (75)	7 (87.5)	6 (75.0)
Glycosylceramides	HexCer	32 (94.1)	31 (91.2)	30 (88.2)
Cholesterol Esters	CE	22 (100)	22 (100)	22 (100)
Monoacylglycerols	MG	11 (91.7)	12 (100)	12 (100)
Diacylglycerols	DG	36 (81.8)	35 (79.5)	37 (84.1)
Triacylglycerols	TG	239 (98.8)	239 (98.8)	238 (98.3)
**Total**		**968 (94.5)**	**970 (95.2)**	**967 (94.9)**

Table S2. Top 20 metabolites with the greatest contribution to PC1 and PC2 from PCA

d1 vs. d0

PC 1		PC 2	
**Metabolite**	**Contribution**	**Metabolite**	**Contribution**
TG 18:1_32:2	0.061897	PG 18:1_20:0	0.068565
TG 18:3_32:1	0.061527	LPI 18:1	0.06856
TG 14:0_36:2	0.060852	PG 18:2_22:0	0.068336
TG 18:1_30:1	0.060808	PA 18:1_20:1	0.067292
TG 18:1_32:3	0.060544	PA 18:0_18:1	0.066549
TG 16:0_34:4	0.06044	TG 20:4_34:2	-0.065192
TG 18:1_34:4	0.059755	TG 16:0_38:5	-0.064518
TG 18:2_30:1	0.05975	PI 18:2_22:1	0.064386
TG 18:1_30:0	0.059654	PI 18:1_22:1	0.064063
TG 18:2_32:2	0.059437	TG 20:4_34:1	-0.062808
TG 14:0_34:2	0.059261	PE P-16:0/16:1	0.062681
TG 14:0_34:1	0.059216	PC O-44:3	0.062562
TG 18:3_30:0	0.058851	PG 18:1_22:1	0.062541
TG 14:0_36:1	0.058606	TG 22:4_34:2	-0.062509
TG 16:0_32:2	0.057961	PI 16:0_22:1	0.06192
TG 14:0_36:3	0.0578	PG 18:2_22:1	0.061782
TG 18:1_33:0	0.057744	PG 18:2_18:2	0.061778
TG 16:0_32:3	0.057607	PI 18:1_18:2	0.06173
TG 20:2_34:2	0.057266	TG 16:0_38:6	-0.061642
TG 18:1_30:2	0.05723	PI 18:0_18:1	0.061595

d3 vs. d0

PC 1		PC 2	
**Metabolite**	**Contribution**	**Metabolite**	**Contribution**
TG 18:1_32:2	0.06218	PA 18:1_18:1	0.08757
TG 18:1_30:1	0.061178	PA 16:1_18:1	0.086
TG 18:1_32:3	0.061078	PG 18:1_18:1	0.085516
TG 14:0_36:2	0.060934	PA 18:1_22:3	0.082229
TG 18:1_30:0	0.06039	PG 16:0_18:1	0.08216
TG 18:2_30:1	0.060389	PG 18:0_18:1	0.081769
TG 14:0_34:1	0.060306	PA 16:2_18:1	0.08111
TG 18:2_32:2	0.06018	PG 16:0_16:1	0.080969
TG 14:0_36:3	0.060015	PA 18:1_20:3	0.080179
TG 18:1_30:2	0.059877	PG 16:3_18:1	0.079541
TG 18:0_34:3	0.059861	PG 18:1_22:0	0.079326
TG 18:2_32:1	0.059628	PG 16:0_20:4	0.079212
TG 16:0_32:2	0.059601	PI 18:1_22:3	0.079194
TG 18:2_30:0	0.059597	PG 17:1_18:1	0.079022

TG 14:0_34:2	0.059515	PI 18:1_20:1	0.078783
TG 16:0_34:4	0.05948	PA 17:1_18:1	0.077929
TG 16:0_32:3	0.059129	PG 16:0_20:3	0.077588
TG 18:0_32:1	0.059078	PA 17:0_18:1	0.077558
TG 18:3_32:1	0.058933	LPG 18:1	0.077551
TG 14:0_36:4	0.058807	PI 18:1_20:0	0.077402

d7 vs. d0

PC 1		PC 2	
**Metabolite**	**Contribution**	**Metabolite**	**Contribution**
TG 18:2_35:1	0.069584	LPE 22:6	0.075757
TG 16:0_36:5	0.069575	MG 20:4	0.074296
TG 18:1_35:3	0.069043	MG 18:1	0.073802
TG 18:1_34:3	0.068838	MG 18:2	0.073158
TG 18:2_34:3	0.068035	PG 16:0_18:2	0.072311
TG 18:2_35:2	0.067996	PG 16:1_18:1	0.071744
TG 18:3_34:2	0.067916	PA 18:1_22:3	0.071698
TG 18:3_34:1	0.067817	PE 36:4	0.070901
TG 16:1_36:4	0.067569	PG 16:0_18:1	0.070648
TG 16:0_37:3	0.067145	MG 16:1	0.070593
TG 18:1_34:4	0.067134	PG 18:0_18:1	0.069361
TG 18:0_36:5	0.067022	PA 16:2_18:1	0.069156
TG 16:0_35:3	0.066817	LPE 22:5	0.069142
TG 18:1_32:2	0.066529	LPE 20:4	0.068981
TG 17:0_36:3	0.066362	PC 32:2	-0.068465
TG 16:0_38:3	0.066265	PC 34:4	-0.068278
TG 18:2_32:1	0.06618	PG 16:0_20:4	0.067731
TG 14:0_36:2	0.066057	PI 18:1_20:1	0.067716
TG 18:2_32:2	0.066055	PA 18:1_18:1	0.067363
TG 16:0_36:4	0.066047	PA 16:0_19:2	0.067044

Table S3. Euclidian distances across all metabolite classes

Metabolite Class	Abbrevation	d1 vs. d0	d3 vs. d0	d7 vs. d0
Phosphatidylethanolamines	PE	1.90	1.81	1.37
Monoacylglycerols	MG	1.90	1.69	1.49
Triacylglycerols	TG	2.06	2.31	0.68
Phosphatidylglycerols	PG	1.15	1.73	2.00
Phosphatidic Acids	PA	1.66	2.06	0.36
Lysophosphatidylethanolamines	LPE	1.26	0.75	1.53
Phosphatidylinositols	PI	1.29	1.52	0.37
Bile Acids	BA	1.54	0.98	0.34
Aminoacids Related	AAR	0.46	0.32	0.79
Acylcarnitines	AC	0.17	0.78	0.62
Aminoacids	AA	0.14	1.22	0.20
Lysophosphatidylinositols	LPI	0.31	0.33	0.79
Fatty Acids	FA	0.34	0.24	0.77
Lysophosphatidylserines	LPS	0.34	0.24	0.73
Ceramides	Cer	0.22	0.33	0.71
Diacylglycerols	DG	0.61	0.11	0.52
Lysophosphatidylglycerols	LPG	0.42	0.22	0.42
Hormones	Hormones	0.10	0.32	0.54
Glycosylceramides	HexCer	0.45	0.26	0.19
Lysophosphatidylcholines	LPC	0.44	0.35	0.04
Biogenic Amines	BioAm	0.17	0.13	0.32
Sphinganines and sphingosines phosphates	SPBP	0.29	0.20	0.11
Carboxylic Acids	CA	0.26	0.15	0.10
Cholesterol Esters	CE	0.07	0.20	0.21
Dihydroceramides	DCer	0.06	0.24	0.16
Indoles Derivatives	ID	0.23	0.02	0.19
Sphinganines and sphingosines	SPBP	0.12	0.21	0.06
Lysophosphatidic Acids	LPA	0.10	0.21	0.09
Sphingomyelins	SM	0.09	0.19	0.11
Phosphatidylcholines	PC	0.07	0.11	0.10
Phosphatidylserines	PS	0.09	0.04	0.13
